# Core Body Temperatures in Intermittent Sports: A Systematic Review

**DOI:** 10.1007/s40279-023-01892-3

**Published:** 2023-08-01

**Authors:** Mitchell J. Henderson, Clementine Grandou, Bryna C. R. Chrismas, Aaron J. Coutts, Franco M. Impellizzeri, Lee Taylor

**Affiliations:** 1 School of Sport, Exercise and Rehabilitation, Faculty of Health, University of Technology Sydney (UTS), Sydney, Australia; 2 Human Performance Research Centre, University of Technology Sydney (UTS), Sydney, Australia; 3 Department of Physical Education, College of Education, Qatar University, Doha, Qatar; 4 School of Sport, Exercise and Health Sciences, Loughborough University, Loughborough, UK

## Abstract

**Background:**

Hyperthermia (and associated health and performance implications) can be a significant problem for athletes and teams involved in intermittent sports. Quantifying the highest thermal strain (i.e. peak core body temperature [peak *T*_c_]) from a range of intermittent sports would enhance our understanding of the thermal requirements of sport and assist in making informed decisions about training or match-day interventions to reduce thermally induced harm and/or performance decline.

**Objective:**

The objective of this systematic review was to synthesise and characterise the available thermal strain data collected in competition from intermittent sport athletes.

**Methods:**

A systematic literature search was performed on Web of Science, MEDLINE, and SPORTDiscus to identify studies up to 17 April 2023. Electronic databases were searched using a text mining method to provide a partially automated and systematic search strategy retrieving terms related to core body temperature measurement and intermittent sport. Records were eligible if they included core body temperature measurement during competition, without experimental intervention that may influence thermal strain (e.g. cooling), in healthy, adult, intermittent sport athletes at any level. Due to the lack of an available tool that specifically includes potential sources of bias for physiological responses in descriptive studies, a methodological evaluation checklist was developed and used to document important methodological considerations. Data were not meta-analysed given the methodological heterogeneity between studies and therefore were presented descriptively in tabular and graphical format.

**Results:**

A total of 34 studies were selected for review; 27 were observational, 5 were experimental (2 parallel group and 3 repeated measures randomised controlled trials), and 2 were quasi-experimental (1 parallel group and 1 repeated measures non-randomised controlled trial). Across all included studies, 386 participants (plus participant numbers not reported in two studies) were recruited after accounting for shared data between studies. A total of 4 studies (~ 12%) found no evidence of hyperthermia, 24 (~ 71%) found evidence of ‘modest’ hyperthermia (peak *T*_c_ between 38.5 and 39.5 °C), and 6 (~ 18%) found evidence of ‘marked’ hyperthermia (peak *T*_c_ of 39.5 °C or greater) during intermittent sports competition.

**Conclusions:**

Practitioners and coaches supporting intermittent sport athletes are justified to seek interventions aimed at mitigating the high heat strain observed in competition. More research is required to determine the most effective interventions for this population that are practically viable in intermittent sports settings (often constrained by many competing demands). Greater statistical power and homogeneity among studies are required to quantify the independent effects of wet bulb globe temperature, competition duration, sport and level of competition on peak *T*_c_, all of which are likely to be key modulators of the thermal strain experienced by competing athletes.

**Registration:**

This systematic review was registered on the Open Science Framework (https://osf.io/vfb4s; 10.17605/OSF.IO/EZYFA, 4 January 2021).

**Supplementary Information:**

The online version contains supplementary material available at 10.1007/s40279-023-01892-3.

## Key Points

**Table:** 

Intermittent sport athletes generate core body temperatures in competition that surpass magnitudes associated with impaired health and performance.
Practitioners and coaches supporting intermittent sport athletes are justified to seek interventions aimed at mitigating the high heat strain observed in competition.
More research is required to determine the independent effects of wet bulb globe temperature, competition duration, sport and level of competition on peak core body temperature—all are likely to be key modulators of the thermal strain experienced by competing athletes.

## Introduction

Heat and temperature affect all biological systems, impacting the successful development, maturation and functioning of even the most basic units of life [1]. In humans, changes of several degrees in core body temperature (*T*_c_) away from a narrow homeostatic range [mean 36.6 °C (95% CI 35.7–37.3 °C)] [2] can be fatal [3, 4]. When working [5] or exercising [6] in hot conditions, heat gain often exceeds loss, allowing heat to accumulate in the body and *T*_c_ to rise. This may lead to hyperthermia and associated reductions in physiological [7] and cognitive performance [8]. Further, increases in *T*_c_ (particularly in combination with dehydration) heighten the risk of exertional heat illness/stroke (EHI/S) [9, 10], which has been proven fatal during occupational pursuits [11], and recreational [12] and professional sport [13]. Large international sporting competitions played in thermally stressful conditions such as World Athletics Championships [14–16], Olympic Games [17–21] and the International Federation of Association Football (FIFA) World Cup [22, 23] have intensified the research interest, as policymakers seek to ensure event safety and athletes/practitioners seek to limit heat-mediated reductions in performance. Understanding the risk and prevalence of high thermal strain and/or hyperthermia in certain sports informs risk mitigation, education and training strategies aimed to protect athlete health and maximise performance.

When athletes perform or train in thermally challenging conditions, they are subject to added physiological strain when compared with the same work in temperate conditions [24–27]. Endurance exercise performance is particularly compromised by high thermal strain (i.e. high *T*_c_) due to cardiovascular adjustments (simultaneously supporting thermoregulation and oxygen delivery), cerebral function changes, muscle metabolism alterations and central nervous system perturbations [28]. In comparison to endurance events, the effects of thermal strain on performance in intermittent sports are less understood (likely due to the complexity of intermittent sport movement patterns and limits in practice/equipment available to athletes and support staff). High-level intermittent sport athletes consistently generate core temperatures above 39 °C during competition regardless of the ambient environmental conditions (*T*_c_ up to 40.5 °C have been observed in athletes when competing in hot conditions [29] and up to 39.8 °C when competing in cool conditions [30]) [31]. Elevation of *T*_c_ is likely to enhance performance in single-sprint events as a result of changes in phosphocreatine utilisation [32], adenosine triphosphate turnover [33] and increased muscle fibre conduction velocity [34]. Despite this, repeated-sprint (< 60 s between efforts) [35, 36], intermittent-sprint (60–300 s between efforts) [37] and neuromuscular performances [38, 39] are impaired when thermal strain is severe (*T*_c_ > 39 °C). These reductions are related to accelerated declines in cardiac output [40], central nervous system output [41], perfusion pressure [42] and blood flow in the exercising muscles (causing greater reliance on anaerobic energy contribution and associated metabolic acidosis) [43]. Work rate during self-paced tasks is also voluntarily reduced under conditions of high thermal stress [41, 44] due to an integrated protective behavioural response governed by effort and thermal perceptions [45]. The combined autonomic and behavioural responses to high thermal strain (and their associated impact on physical performance) potentially endanger the health and competition outcomes for competing intermittent sport athletes.

In addition to the physical impairments resulting from excessively high *T*_c_, heat strain can also compromise cognitive function [46] and exacerbate mental fatigue [47]. Cognition supports decision-making in sport as athletes are required to process task-specific information from their competitive environment and match sensory inputs with an appropriate action [48]. Although small improvements in cognitive function are common after moderate *T*_c_ increases (potentially due to increased arousal [49] or cerebral blood flow [50]) [51–53], severe thermal strain has been observed to impair cognitively complex task performance in occupational [53–55] and athletic populations [56]. The current theory suggests that this is related to both hyperthermia and cognitive tasks competing for finite cerebral resources (cortical activity [38, 57] and output intensity from the prefrontal cortex [58, 59]), and performance declines when these capacities are overloaded by complex tasks [54]. High heat strain decreases vigilance and reaction test performance [60], and increases perceptions of fatigue and discomfort [61, 62], frequency of unsafe behaviours [63] and error rates in a visual–motor tracking test [64], flight simulators [65] and pilots in flight [66]. Mental fatigue resulting from hyperthermia can lead to further reductions in tactical performance [67] and has also been shown to impair technical skill execution [68]—both accepted constructs of intermittent sports performance [69, 70] and key differentiators of success [71, 72]. Combined, these cognitive impairments have the potential to threaten the health and performance of athletes in competition (where execution of cognitively complex tactical decision-making and technically complex skills is frequent and has a large influence on match outcomes [73]).

Hyperthermia (and associated health and performance implications) can be a significant problem for athletes and teams involved in intermittent sports. Quantifying the highest thermal strain (i.e. peak *T*_c_) from a range of intermittent sports would enhance our understanding of the thermal requirements of sport and assist in making informed decisions about training or match-day interventions to reduce thermally induced harm and/or performance decline. The efficacy of applied heat acclimation/acclimatisation training interventions [27] and acute mixed-method cooling protocols [21] is supported by a considerable body of evidence [74]. Therefore, with increasing globalisation in sport enabling year-round competition in warmer climates and the ongoing effects of climate change [75], best practice management of exercise-induced hyperthermia (through targeted application of these interventions) will be of increasing importance. Identifying appropriate action for athletes and support staff should be informed by available peer-reviewed literature, and currently, no reviews of the literature provide a synthesis of the thermal strain data collected in-competition during intermittent sports. Further, increased understanding of the magnitude of thermal strain in competing athletes could be used to guide policy surrounding thermoregulatory health and safety at sporting events. We, therefore, systematically reviewed the literature investigating athletes’ peak *T*_c_ during competition in a variety of intermittent sports. The purpose of this review is to provide athletes, practitioners and policy-makers a synthesis of the thermal strain literature to determine the need for interventions aimed at mitigating exercise-induced hyperthermia in intermittent sport athletes.

## Methods

This review was conducted and reported according to Preferred Reporting Items for Systematic Reviews and Meta-Analyses (PRISMA) guidelines [76]. A systematic review protocol that included the review question, search strategy and exclusion criteria was registered with the Open Science Framework (https://osf.io/vfb4s; 10.17605/OSF.IO/EZYFA, 4 January 2021).

### Eligibility Criteria

Eligibility criteria were drafted and subsequently refined by three authors (MH, FMI and LT) using a random sample of studies. For this review, intermittent sport was operationally defined as all sports characterised by intermittent bursts of high-intensity exercise and requiring the execution of complex sport-specific skills and cognitive tasks over a more prolonged period (minutes to hours), with longer breaks at scheduled intervals (e.g. quarters, half time) as well as unscheduled times (e.g. injury or restarting play after scoring in soccer or rugby) [77]. Studies were considered eligible if they included healthy athletes competing in intermittent sports competition at any level. Non-human subjects, youth athletes (study participants’ mean age minus 2 standard deviations is less than 16 years) or participants with chronic disease, disability, metabolic disorders or injury were excluded due to differences in the physiological responses to sports. Interventions aimed at both adult and youth athletes were included only if the data provided for adults were reported separately. All exposures including athletes involved in intermittent sport competition (competitive, friendly or experimental) played within normal parameters (e.g. field size, playing numbers) were included in this review. Outcomes of interest included only internally measured *T*_c_ (i.e. gastrointestinal or rectal; shown to display acceptable agreement [78]). No limitations were placed on the study design if the intervention met the eligibility criteria. Studies were included only if *T*_c_ was measured during competition or breaks in play and without experimental intervention that may influence thermal strain (e.g. cooling).

### Search

A literature search was conducted by one author (MH) in the electronic bibliographic databases of Web of Science Core Collection, Ovid MEDLINE and EBSCOhost SPORTDiscus. Databases were searched from inception up until April 2023. No language or publication status restrictions were imposed on the search to ensure literature saturation. Literature search strategies were developed using search terms related to *T*_c_ measurement and intermittent sports competition. The keywords were derived using the {litsearchr} package [79] in R statistical software [80], as has been described previously [81]. The {litsearchr} package uses text mining and keyword co‐occurrence networks to efficiently identify potential keywords without relying on a potentially biased set of preselected articles, resulting in the development of a partially automated and systematic search strategy [81]. The code used to derive the keywords and Boolean search string are available (https://osf.io/xam5v), and an explanation of the method/code is provided in Supplementary Material 1. The Boolean search string used on all databases with results is provided in Supplementary Material 2. Trial/study registries were searched during a pilot phase; however, due to the non-clinical nature of this review, no results were found. In conjunction with the database searches, the reference lists of relevant studies, reviews and books were screened for possible omissions. Relevant experts in the field were also consulted, and their profiles were searched to ensure saturation of the literature.

### Study Selection

Articles retrieved through the systematic search were exported into a reference management software (EndNote version X8), and all duplicate articles were removed. All references were then imported into Covidence (Covidence Systematic Review Software, Veritas Health Innovation, 2013) for assessment of eligibility. Two authors (MH and CG) independently screened the records by title and abstract, with all potentially eligible references proceeding to full-text screening, with conflicts resolved by a third author (FMI). Authors (MH and CG) then independently screened the full text of all included articles against the eligibility criteria. Interrater reliability, as measured by Cohen’s Kappa (*κ*), was 0.75 during the title and abstract screening and 0.85 during full-text screening.

### Data Extraction

Data were extracted by two authors (MH and CG) and imported into an Excel spreadsheet created for this review (Supplementary Material 3). Extracted data were compared, with any discrepancies resolved through discussion. Information extracted from each eligible study included publication details (author and year), participant characteristics (sex, level of competition and sample size), study methods (design, types of measurement and recording frequency), exposure (sport, competition type, duration, environmental conditions, location of data collection and home location of participants) and outcome (*T*_c_).

### Data Synthesis

Data were not meta-analysed given the methodological heterogeneity between studies (including differing core temperature measurements, environmental conditions and exposure durations to a variety of sports). We anticipated, based on a scoping search, that the heterogeneity could not be explored given that subgroup analyses would leave too few studies in each group for investigating the different moderators. Data were therefore presented descriptively in tabular and graphical format. Although a meta-analysis was not planned (or pre-registered) for the aforementioned reasons, summary estimates and forest plots of overall and subgroup meta-analyses have been provided at the request of a reviewer (Supplementary Material 4). As expected, high heterogeneity was found in both the overall and subgroup analyses.

Mean *T*_c_ for each study condition was calculated with confidence intervals (50%, 80%, 95% and 99%) and compared with homeostatic [2] and hyperthermic [28] ranges. Standard deviations for *T*_c_ (reported in the text or extracted from figures) were converted to standard errors by dividing by the square root of the sample size. Standard errors were subsequently converted to confidence intervals by multiplying by the *Z*-value associated with the desired level of confidence (*Z* = 0.674, 1.282, 1.960 and 2.576 for 50%, 80%, 95% and 99% confidence intervals, respectively). Finally, adding or subtracting the resulting values from the mean provided upper or lower confidence limits. All studies and group conditions that reported parametric measures of centrality and variability (mean and standard deviation) were included in this synthesis. Medians and interquartile ranges reported in Stay et al. [82] were transformed to estimated means and standard deviations using the method outlined in Luo et al. [83]. Mean values without standard deviations reported in Blanksby et al. [84] were included but without confidence intervals. This synthesis, therefore, includes data from 38 group conditions and all included studies.

A secondary synthesis was performed that examined the relationship between competition duration, wet bulb globe temperature (WBGT), number of observations and peak *T*_c_ between study groups. All studies that provided (1) competition duration and (2) either WBGT or ambient temperature and relative humidity were considered eligible for this synthesis. Nine studies did not report WBGT, so estimates were calculated using the validated Liljegren method [85]. In the case of a maximum WBGT threshold being reported (e.g. < 18 °C), the upper limit was used. In the case of a WBGT range being reported (minimum to maximum), the midpoint of the range was used. Data for Stay et al. [82] could not be included due to not reporting an absolute competition duration. Data from Delamarche et al. [86], Kouassi et al. [87], Mohr et al. [88] and Pliauga et al. [89] also could not be included due to insufficient environmental data being reported to calculate a WBGT estimate. Attempts were made to contact the authors in each of these cases, but additional data was not able to be obtained. This secondary synthesis, therefore, includes data from 31 group conditions from 29 out of 34 (~ 85%) included studies. In both analyses, when shared data were used between studies, the study that reported the largest sample size for a given condition was used to prevent duplicate data from influencing results.

### Risk of Bias Assessment

The current review focused on a specific outcome (*T*_c_) as measured in a control (non-experimentally manipulated) condition. However, no available tool, to our knowledge, specifically includes potential sources of bias for physiological responses in such a (descriptive) context. We, therefore, did not examine the risk of bias but instead developed and used a methodological evaluation checklist to document what we deemed to be important methodological considerations for researchers conducting future investigations.

#### Methodological Evaluation Checklist

A. Clearly described population (age, sex and level of competition).
B. Environmental information reported [wet bulb globe temperature (WBGT) or ambient temperature and relative humidity; must be on a continuous scale and not made discrete, such as < 18 °C].
C. Information regarding the inclusion of substitutes or time spent out of competition for participants reported (if sport includes substitutes).
D. Stating the duration of gastrointestinal device ingestion before measurement period (when a gastrointestinal device is used).
E. Control of cold or hot food/beverage consumption during the measurement period (if the duration of gastrointestinal pill ingestion is less than 5 h).
F. Reporting of menstrual cycle phase in female athletes.
G. Continuous measurement of core temperature (as opposed to discrete time points such as pre and post-match).
H. Reporting tests/checks for normality of data.
I. Missing data addressed and justified (if present).

## Results

### Study Selection

**Fig. 1:**
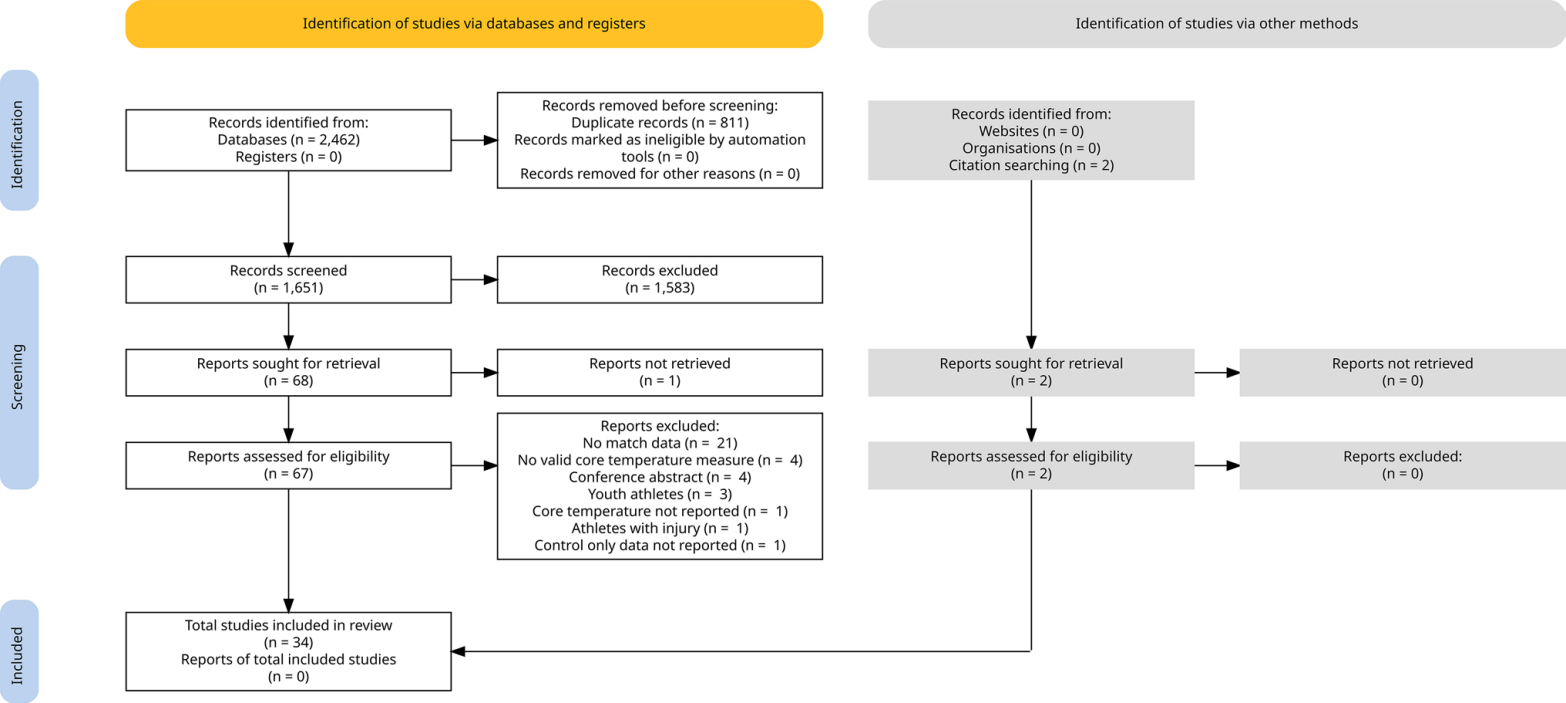
PRISMA flow diagram of systematic search and included studies

The initial database search yielded 2462 studies. Once duplicates were removed, 1651 titles and abstracts were screened for inclusion, and of those, 1583 studies were excluded based on the eligibility criteria. A total of 68 studies were retrieved as full text and assessed for eligibility (1 report not retrieved [90]), and of those, 35 were excluded (reasons for the exclusion provided in Fig. 1 and Supplementary Material 5). An additional two studies that met the inclusion criteria were identified by searching reference lists. Upon completion of these procedures (Fig. 1), 34 studies were included for analysis in this systematic review (Table 1).

**Table 1:** Descriptive results and characteristics of included studies

Study	Study design	Participants	Sport	Level of competition	Measurement type	Activity	Environmental conditions	Peak core body temperature
Aughey et al. [29]	Observational	35 M	Australian Rules football	Level 5	Gastrointestinal	8 hot matches (HOT) 8 cool matches (COOL) (friendly and competitive)	WBGT: HOT = 28 °C (3); COOL = < 18 °C Ambient temperature: HOT = 27 °C (2); COOL = 17 °C (4) Relative humidity: HOT: 58% (15); COOL: 51% (11)	HOT: 39.5 °C (0.5) COOL: 39.4 °C (0.6)
Blanksby et al. [84]	Observational	27 M	Squash	Levels 1, 2 and 3	Rectal	27 experimental matches (9 matches of each 3 playing standards)	Ambient temperature: < 22.2 °C Relative humidity: < 60%	A grade: 39.1 °C Active: 38.9 °C Sedentary: 38.9 °C
Cohen et al. [117]	Observational	15 M	Rugby union	Level 2	Rectal	1 competitive match	Ambient temperature: 24.5 °C Relative humidity: 31%	39.4 °C (0.5)
Dancaster [98]	Observational	Not reported	Rugby union	Level 2	Rectal	5 competitive matches	WBGT: 20.5 °C (2.7) Ambient temperature: 19–34.5 °C	39.6 °C (0.8)
Delamarche et al. [86]	Observational	6 M	Handball	Level 5	Rectal	1 experimental match	Ambient temperature: 18–20 °C	39.4 °C (0.3)
Diaw et al. [118]	Experimental (parallel design RCT)	11 M (control group only)	Soccer	Level 2	Rectal	2 experimental matches	Ambient temperature: 24.5–25 °C Relative humidity: 65–68%	37.3 °C (0.6)
Duffield et al. [109]	Quasi-experimental (repeated measures design NRCT)	7 M (control match only)	Soccer	Level 5	Gastrointestinal	1 competitive match (recorded over a period of 5 matches)	WBGT: 26 °C (2) Ambient temperature: 27 °C (2) Relative humidity: 80% (10)	39.9 °C (0.4)
Duffield et al. [110]	Observational	10 M	Australian Rules football	Level 5	Gastrointestinal	2 friendly matches	WBGT: 27.6 °C (2.3) Ambient temperature: 29.5 °C (1.3) Relative humidity: 64.9% (16.7)	39.5 °C (0.4)
Edwards and Clark [108]	Observational	15 M	Soccer	Levels 2 and 5	Gastrointestinal	2 friendly matches (1 recreational; 1 professional)	Ambient temperature: 16 °C (recreational) and 19 °C (professional) Relative humidity: 47% (recreational) and 53% (professional)	Recreational: 39.4 °C (0.5) Professional: 38.8 °C (0.4)
Elliot et al. [116]	Observational	8 M	Tennis	Level 3	Rectal	4 experimental matches	WBGT: 21.5 °C (1.9) Ambient temperature: 22.7 °C (1.8) Relative humidity: 64.3% (7.2)	38.5 °C (0.4)
Fenemor et al. [106]	Observational	11 M	Rugby sevens	Level 5	Gastrointestinal	5 competitive matches	Mean across all 5 matches: Ambient temperature: 29.3 °C Relative humidity: 75% WBTp: 25.3 °C	39.0 °C (0.4)
Girard et al. [93]	Observational	12 M	Tennis	Level 4	Rectal	2 experimental matches each [1 in hot conditions (HOT); 1 in cool conditions (COOL)]	WBGT: HOT = 33.6 °C (0.9); COOL = 19.4 °C (0.3) Ambient temperature: HOT = 36.8 °C (1.5); COOL = 21.8 °C (0.1) Relative humidity: HOT = 36.1% (11.3); COOL = 72.3% (3.2)	HOT: 39.4 °C (0.5) COOL: 38.7 °C (0.2)
Girard et al. [92]	Observational	12 M	Tennis	Level 4	Rectal	2 experimental matches each [1 in hot conditions (HOT); 1 in cool conditions (COOL)]	WBGT: HOT = 33.6 °C (0.9); COOL = 19.4 °C (0.3) Ambient temperature: HOT = 36.8 °C (1.5); COOL = 21.8 °C (0.1) Relative humidity: HOT = 36.1% (11.3); COOL = 72.3% (3.2)	HOT: 39.4 °C (0.5) COOL: 38.7 °C (0.2)
Goodman et al. [99]	Observational	Not reported	Rugby union	Level 2	Rectal	3 competitive matches	Ambient temperature: 17.8–22.6 °C Relative humidity: 18–85%	39.2 °C (0.4)
Henderson et al. [30]	Observational	12 F	Rugby sevens	Level 5	Gastrointestinal	3 competitive matches	WBGT: 18.9–20.1 °C	39.2 °C (0.5)
Hornery et al. [112]	Observational	14 M	Tennis	Level 4	Gastrointestinal	33 competitive matches (2 hard court tournaments; 1 clay tournament)	Ambient temperature: Hard court = 32.0 (4.5); Clay = 25.4 (3.8) Relative humidity: Hard court = 38 (14); Clay = 32 (5)	Hard court: 38.9 (0.3) Clay: 38.5 (0.6)
Huang et al. [113]	Experimental (repeated measures design RCT)	10 M	Baseball	Level 3	Rectal	2 experimental matches	Ambient temperature: 31.1–33.4 °C Relative humidity: 63–67%	38.15 °C (0.31)
Knez and Périard [97]	Observational	10 M	Tennis	Level 4	Rectal	2 experimental matches each [1 in hot conditions (HOT); 1 in cool conditions (COOL)]	WBGT: HOT = 33.6 °C (0.9); COOL = 19.5 °C (0.3) Ambient temperature: HOT = 36.7 °C (1.6); COOL = 21.8 °C (0.1) Relative humidity: HOT = 35.9% (11.9); COOL = 73.3% (2.9)	HOT: 39.3° (0.5) COOL: 38.7° (0.2)
Kouassi et al. [87]	Quasi-experimental (parallel NRCT)	20 F and M (control group only)	Judo	Level 4	Rectal	20 competitive bouts	Not reported	38.3 (0.3)
Mohr et al. [88]	Experimental (parallel design RCT)	16 M Control (*n* = 8) Re-warmup (*n* = 8)	Soccer	Level 4	Rectal	1 experimental match	Not reported	Control: 38.9 (0.1) Re-warmup: 39 (0.2)
Mohr et al. [119]	Experimental (repeated measures design RCT)	17 M	Soccer	Unclear	Gastrointestinal (7) and rectal (10)	2 experimental matches [1 in hot conditions (HOT); 1 in control conditions (CON)]	Ambient temperature: HOT = 43 °C; CON = 21 °C Relative humidity: HOT = 12%; CON = 55%	HOT: 39.7 (0.41) CON: 38.8 (0.82)
Morante and Brotherhood [102]	Observational	6 F 19 M	Tennis	Levels 2 and 4	Rectal	47 experimental matches	WBGT: 22.5 °C (4.3) Ambient temperature: 25.0 °C (5.4) Relative humidity: 50.7% (14.3)	38.72 (0.38)
Morante and Brotherhood [100]	Observational	6 F and M	Tennis	Level 2	Rectal	6 experimental matches	Not reported	38.31 °C
Morante and Brotherhood [101]	Observational	6 F 19 M	Tennis	Level 2	Rectal	43 experimental matches	WBGT: elite [F = 24.4 °C (4.9), M = 23.0 °C (3.0)]; recreational [F = 20.9 °C (6.2), M = 22.1 °C (4.7)] Ambient temperature: elite [F = 26.9 °C (6.4), M = 25.0 °C (3.8)]; recreational [F = 23.3 °C (7.1), M = 24.9 °C (6.4)]	Elite: F = 38.4 °C (0.3); M = 38.5 °C (0.4) Recreational: F = 38.2 °C (0.3); M = 38.4 °C (0.4)
Naito et al. [114]	Experimental (repeated measures design RCT)	4 F 4 M	Tennis	Level 3	Gastrointestinal	1 experimental match	WBGT: 30.9 °C (1.2)	38.6 °C (0.7)
Ozgunen et al. [111]	Observational	10 M	Soccer	Level 4	Gastrointestinal	2 experimental matches	Ambient temperature: Match 1 = 34 °C (1); Match 2 = 36 °C (0) Relative humidity: Match 1 = 38% (2); Match 2 = 61% (1)	Match 1: 39.1 °C (0.4) Match 2: 39.6 °C (0.3)
Périard et al. [94]	Observational	10 M	Tennis	Level 4	Rectal	2 experimental matches each [1 ad libitum fluid consumption (HOT); 1 with hydration plan (HYD)]	WBGT: HYD = 35.2 °C (2.4); HOT = 34.2 °C (0.4) Ambient temperature: HYD = 36.9 °C (2.3); HOT = 36.8 °C (0.3) Relative humidity: HYD = 32.5% (12.8); HOT = 33.3% (3.8)	HOT: 39.4 °C (0.5) HYD: 39.2 °C (0.6)
Périard et al. [95]	Observational	12 M	Tennis	Level 4	Rectal	2 experimental matches each [1 in hot conditions (HOT); 1 in cool conditions (COOL)]	WBGT: HOT = 33.6 °C (0.9); COOL = 19.4 °C (0.3) Ambient temperature: HOT = 36.8 °C (1.5); COOL = 21.8 °C (0.1) Relative humidity: HOT = 36.1% (11.3); COOL = 72.3% (3.2)	HOT: 39.4 °C (0.5) COOL: 38.7 °C (0.2)
Périard et al. [96]	Observational	12 M	Tennis	Level 4	Rectal	2 experimental matches each [1 in hot conditions (HOT); 1 in cool conditions (COOL)]	Ambient temperature: HOT = 36.8 °C (1.5); COOL = 21.8 °C (0.1) Relative humidity: HOT = 36.1% (11.3); COOL = 72.3% (3.2)	HOT: 39.4 °C (0.5) COOL: 38.7 °C (0.2)
Pliauga et al. [89]	Observational	10 M	Basketball	Level 3	Rectal	1 experimental match	Not reported	39.4 °C (0.4)
Stay et al. [82]*	Observational	38 M	Cricket	Level 4	Gastrointestinal	6 competitive 4-day matches	WBGT: Batters = 23.7 (IQR: 15.6–31.8); fielders = 24.2 (IQR: 17.0–31.4) Ambient temperature: Batters = 27.6 (range: 22.4–32.8); fielders = 27.7 (range: 20.9–34.5) Relative humidity: Batters: 52.7 (range: 35.6–69.8); fielders: 48.1 (range: 34.9–61.3)	Batters: 38.5 (IQR: 37.7—39.3) Fielders: 38 (IQR: 37.3—38.7)
Taylor et al. [91]	Observational	17 M	Rugby sevens	Level 5	Gastrointestinal	11 competitive matches	WBGT: 22.1 °C (4.9)	38.5 °C (0.6)
Tippet et al. [107]	Observational	7 F	Tennis	Level 5	Gastrointestinal	7 competitive matches	WBGT: 30.3 °C (2.3)	39.13 °C (0.34)
Veale and Pearce [115]	Observational	15 M	Australian Rules football	Level 3	Gastrointestinal	4 friendly matches	WBGT: Day 1 = 33–33.9 °C; Day 2 = 20.5–24.8 °C Ambient temperature: Day 1 = 25–31.4 °C; Day 2 = 20.1–26.8 °C Relative humidity: Day 1 = 42–66%; Day 2 = 47–63.7 °C	39 °C (0.2)

*RCT* randomised controlled trial, *NRCT* non-randomised controlled trial, *M* male, *F* female, *WBGT* wet bulb globe temperature, *WBTp* psychrometric wet bulb temperature, *IQR* interquartile range

*Reported median and interquartile range

### Characteristics of the Publications

**Fig. 2:**
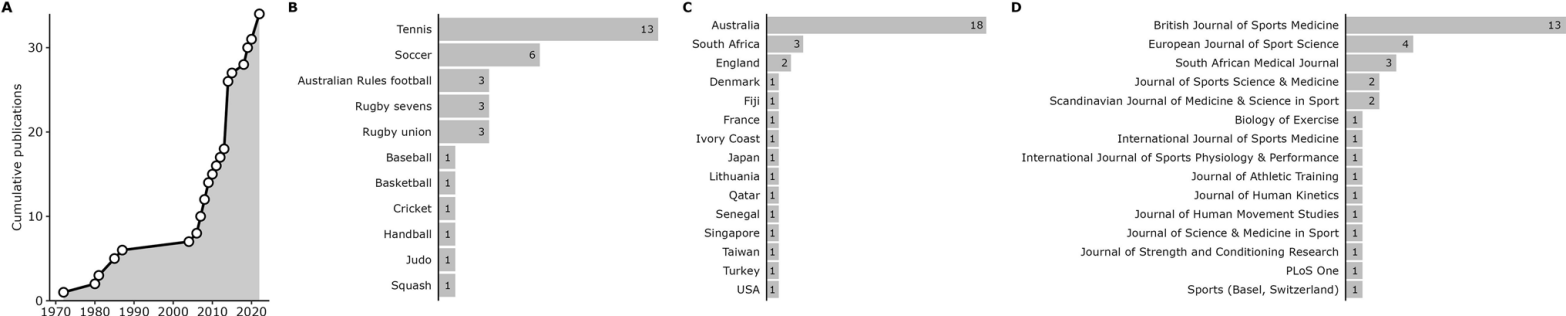
Publication characteristics of the included studies. Cumulative number of publications over time (**A**), count of included studies by sport (**B**), country (**C**) and by journal (**D**)

The studies were published between 1972 and 2022 (Fig. 2A) in 11 different sports (Fig. 2B), 15 different countries (Fig. 2C; one study was conducted across two countries [91] and has been included in counts for both) and 15 different peer-reviewed journals (Fig. 2D). Six studies were published in a *British Journal of Sports Medicine* supplement focused on heat stress and tennis performance in April 2014 [92–97]. The key characteristics of each study are presented in Table 1.

### Characteristics of the Participants

Across all included studies, 386 participants (plus no reported participant numbers for two studies [98, 99]) were recruited after accounting for shared data between studies [92–97, 100–102]. Male-only participants were involved in 27 of the included studies (~ 79%), whilst only 2 studies included female-only participants (~ 6%). Four studies included a combination of female and male participants (~ 12%), and one study did not report the sex of their participants (~ 3%). There were no consistent classifications to describe the participant’s level of competition among the included studies, so the five-level classification system defined by Russell et al. [103] (adapted from De Pauw et al. [104] and Decroix et al. [105]) was used. Classifications are:

- Level 1: Untrained or sedentary.
- Level 2: Habitually active, physically fit and recreationally trained.
- Level 3: Trained and competitive; high-level youth competition.
- Level 4: Highly trained and competitive; semi-professional athletes.
- Level 5: Professional; full-time paid athletes in professional competitive leagues.

Using these defining criteria, nine studies examined level 5 participants (~ 26%) [29, 30, 86, 91, 106–110], 12 studies examined level 4 participants (~ 35%) [82, 87, 88, 92–97, 102, 111, 112], 6 studies examined level 3 participants (~ 18%) [84, 89, 113–116], 9 studies examined level 2 participants (~ 26%) [84, 98–102, 108, 117, 118] and 1 study examined level 1 participants (~ 3%) [84]. One study did not clearly report their participants level of competition [119]. Three investigations included participants from more than one level [84, 102, 108], and these studies have been included in counts for each level of participant examined within the study.

### Study Characteristics

**Fig. 3:**
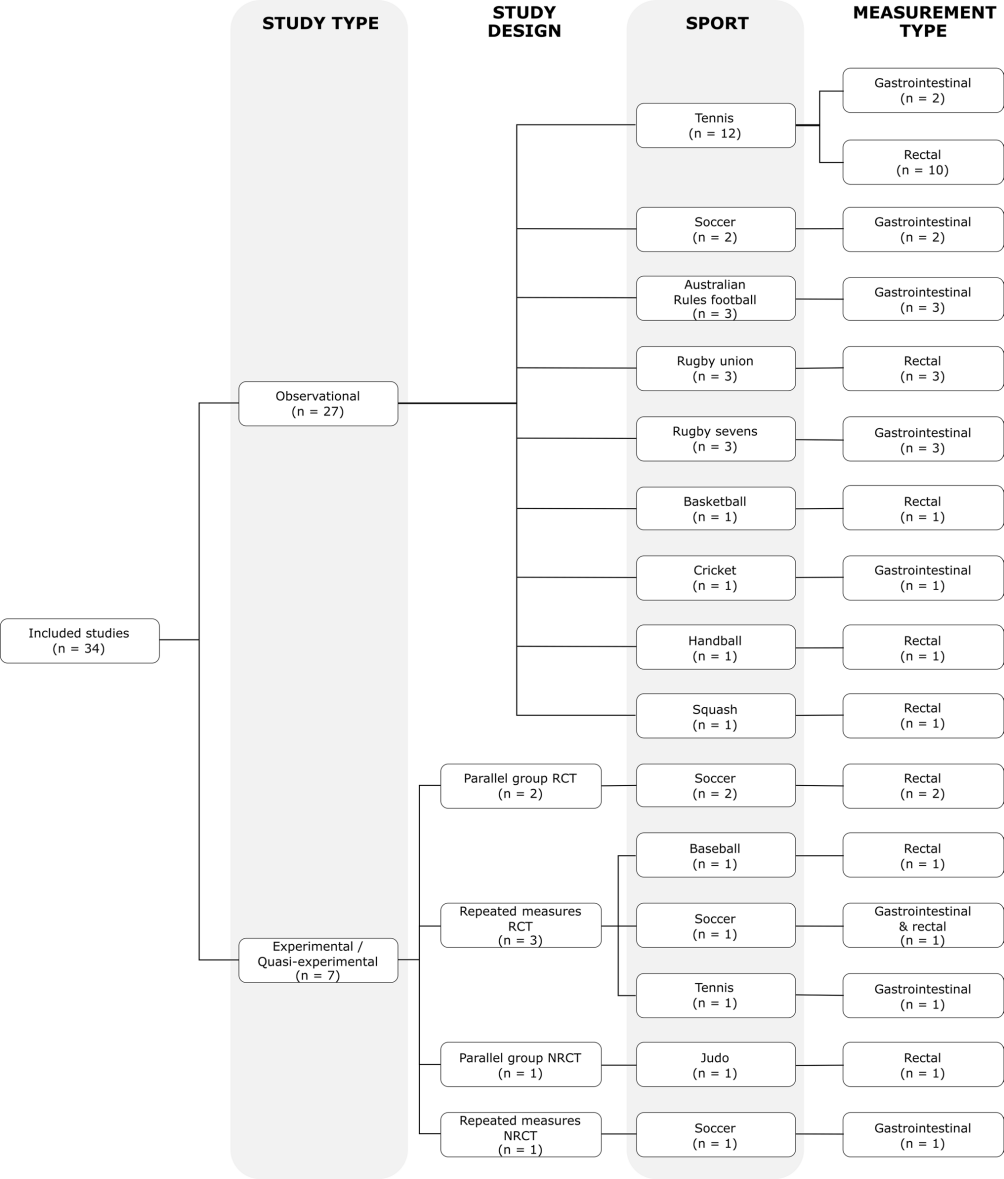
Flow diagram comparing the study types, designs, sports and measurement types of the included studies. *RCT* randomised controlled trial, *NRCT* non-randomised controlled trial

Among the 34 included studies, 27 were observational, 5 were experimental (2 parallel group and 3 repeated measures randomised controlled trials), and 2 were quasi-experimental (1 parallel group and 1 repeated measures non-randomised controlled trial). In-competition *T*_c_ has been reported in tennis [92–97, 100–102, 107, 112, 114, 116], soccer [88, 108, 109, 111, 118, 119], rugby union [98, 99, 117], Australian Rules football [29, 110, 115], rugby sevens [30, 91, 106], squash [84], judo [87], handball [86], cricket [82], basketball [89] and baseball [113]. Rectal measures of *T*_c_ were most common and used in 21 studies (~ 62%), whereas gastrointestinal methods were used in 14 studies (~ 41%; one study was forced to use a combination due to technical difficulties with their gastrointestinal devices and thus has been counted in both). A comparison of the study designs, sports and measurement types of the included studies is included in Fig. 3.

### Core Body Temperature

**Fig. 4:**
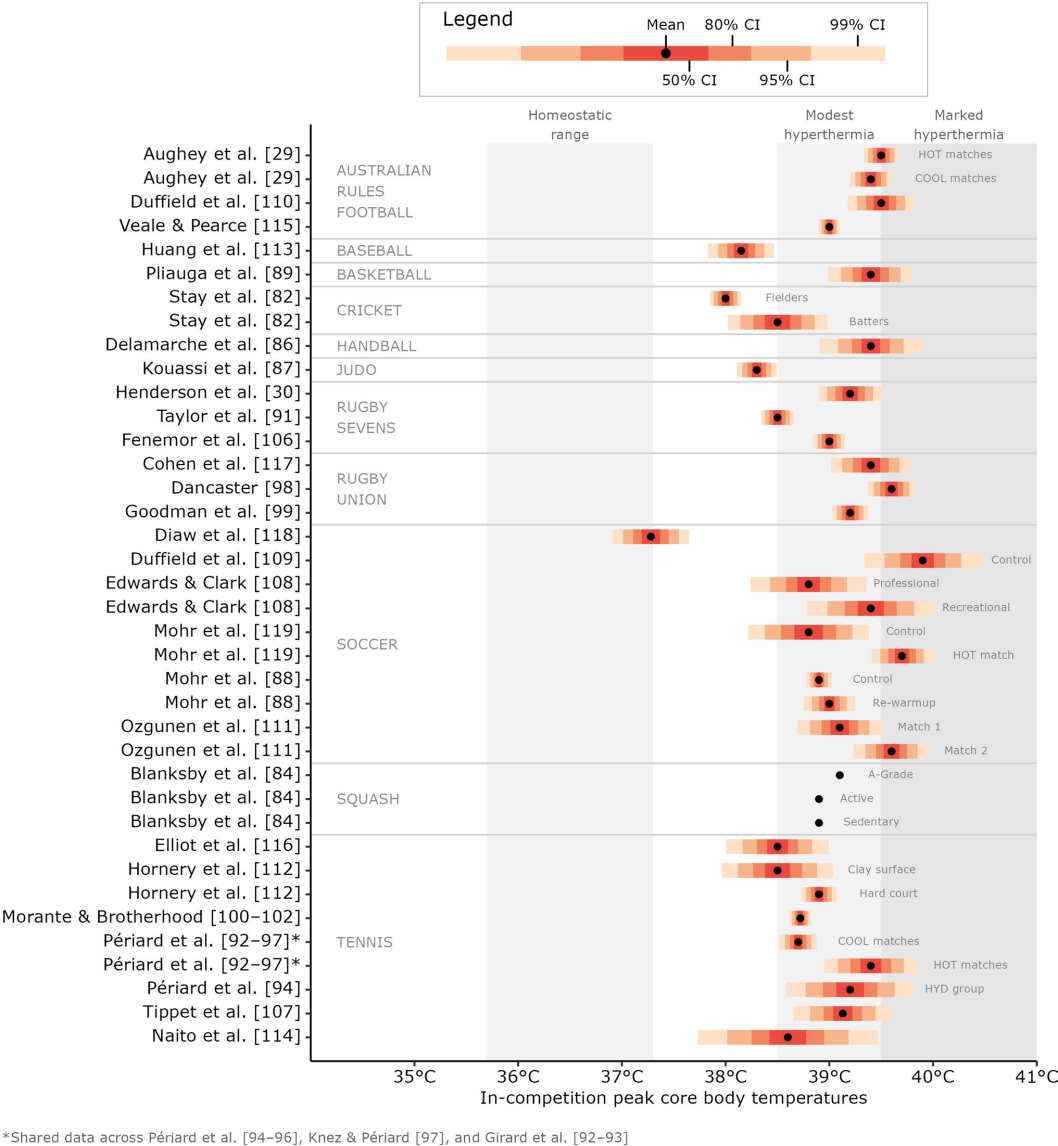
Peak core body temperatures measured in competition during different intermittent sports. Black circles represent the group mean, and the coloured bands represent levels of confidence. Grey text to the right of the data shows the study group being represented when a study reports multiple groups. Grey-shaded areas represent the homeostatic [2] and hyperthermic [28] ranges of core body temperature and are individually labelled above. Data shared across multiple studies are only represented once. Confidence intervals could not be constructed for the Blanksby et al. [84] data due to no measure of variability being reported; hence, the mean value is presented alone. *CI* confidence interval

**Fig. 5:**
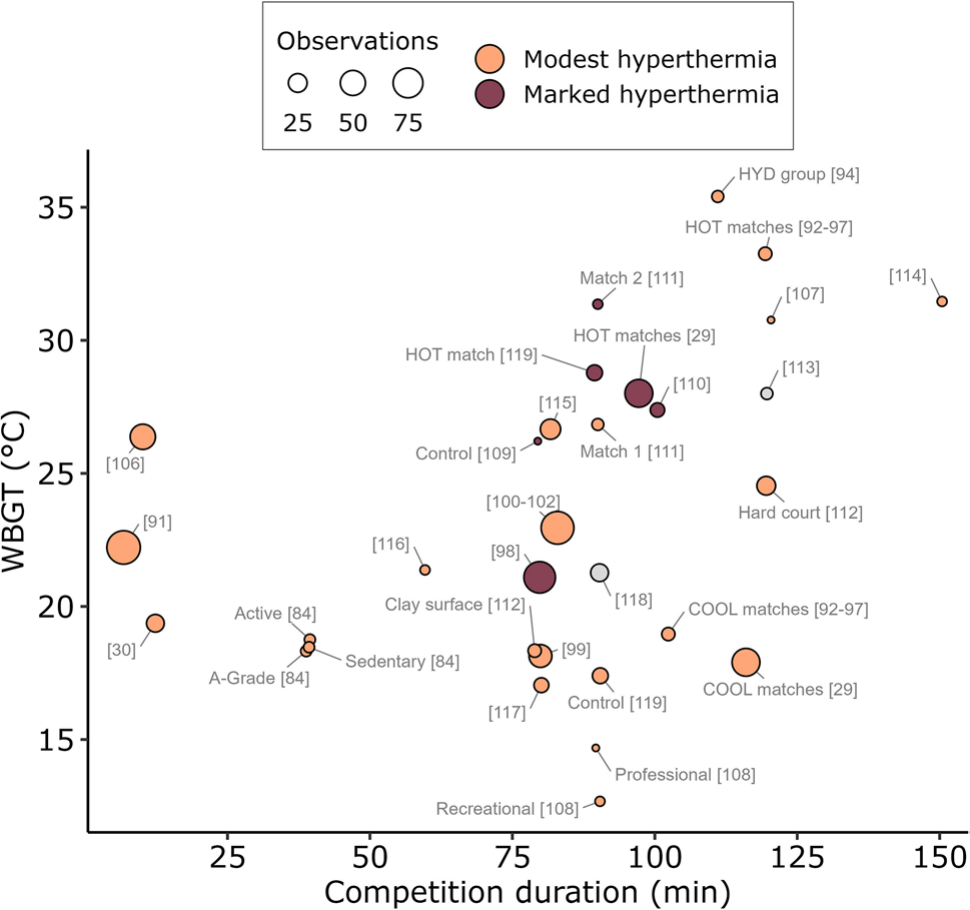
Relationship between competition duration, wet bulb globe temperature (WBGT), number of observations (within group sample size) and peak core body temperature (peak *T*_c_). Where studies did not report WBGT, estimates based on the Liljegren method [85] were calculated. Peak *T*_c_ values between 38.5 and 39.5 °C are classified as modest hyperthermia and 39.5 °C or greater as ‘marked’ hyperthermia [28]. Data shared across multiple studies are only represented once. To uncover overlapping data points, a small amount of random variation to the location of each point was applied (known as jittering). *WBGT* wet bulb globe temperature, *HYD* experimental group following a hydration regimen

Of the 34 studies included in this systematic review, 4 (~ 12%) found no evidence of hyperthermia, 24 (~ 71%) found evidence of ‘modest’ hyperthermia (between 38.5 and 39.5 °C [28]), and six (~ 18%) found evidence of ‘marked’ hyperthermia (39.5 °C or greater [28]) during intermittent sports competition (Fig. 4). All 13 studies examining tennis athletes in competition found modest hyperthermia. Three of the six investigations on soccer athletes during play detected marked hyperthermia, and two detected modest hyperthermia. Of the three studies conducted on Australian Rules football athletes, two found marked hyperthermia, and the other found modest hyperthermia. One study in rugby union found marked hyperthermia, whilst the other two rugby union and all three rugby sevens studies found modest hyperthermia. In the single studies examining basketball, cricket (batters), handball and squash, each found modest hyperthermia on average during play. The studies where no hyperthermia was found were baseball, cricket (fielders), judo and soccer. The available WBGT values across all studies that found some degree of hyperthermia ranged from < 18 to 35.2 °C, and available exposure times (competition duration) ranged from a 5-min judo bout (not included in the figure as environmental conditions were not reported) to tennis matches lasting 2–3 h (Fig. 5).

### Methodological Evaluation

The methodological evaluation results from the included studies are available in Table 2. Most included studies in this systematic review clearly described their study population (30 out of 34 studies; ~ 88%) and adequately reported the environmental conditions during their data collection period (28 out of 34 studies or ~ 82%; although increased reporting of WBGT, rather than ambient temperature and relative humidity, would facilitate more standardised comparison and more valid statistical models to be produced). Whether interchange players were used was reported in 8 out of 17 studies examining sports that include substitutions (47%), potentially influencing group mean values when athletes with lower exposure durations are grouped with athletes completing a full match or bout. Of the 14 studies using gastrointestinal *T*_c_ measurements, 12 (86%) adequately reported the ingestion time before data collection (required to make assessments on the validity of the measurement [120]). When less than 5 h had elapsed between gastrointestinal device ingestion and data collection, which was the case for seven included studies, only three (43%) reported controlling for nutrition and hydration. None of the seven investigations including female participants reported their menstrual cycle phase. Missing data were found in 11 included studies, but only in 8 (73%) of these were the missing data reported with appropriate justification provided. Very few studies included in this systematic review included continuous *T*_c_ monitoring over the data collection period (7 out of 34 studies; ~ 21%), likely due to technology advancements only making this readily accessible in applied settings within the last decade. Similarly, the prevalence of reporting the results of tests and checks of normality before parametric statistical analysis was very low (6 out of 34 studies; ~ 18%).

**Table 2:** Methodological evaluation checklist results

Study	Year	Clearly described population	Environmental information reported	Reported whether substitutes were used (including time spent in/out of competition)	Ingestion time prior to data collection reported (when gastrointestinal measurement used)	Control of nutrition consumption (if < 5 h post-ingestion)	Menstrual cycle phase reported	Continuous data measurement	Reporting tests/ checks for normality of data	Missing data addressed and justified (if present)
Aughey et al. [29]	2014	+	+	+	+	−	N/A	−	−	N/A
Blanksby et al. [84]	1980	+	−	N/A	N/A	N/A	N/A	−	−	N/A
Cohen et al. [117]	1981	+	+	−	N/A	N/A	N/A	−	−	+
Dancaster [98]	1972	−	+	−	N/A	N/A	N/A	−	−	N/A
Delamarche et al. [86]	1987	+	−	−	N/A	N/A	N/A	−	−	−
Diaw et al. [118]	2014	+	+	+	N/A	N/A	N/A	−	−	N/A
Duffield et al. [109]	2013	+	+	+	+	N/A	N/A	−	−	+
Duffield et al. [110]	2009	+	+	−	+	N/A	N/A	−	−	−
Edwards and Clark [108]	2006	+	+	−	+	+	N/A	−	−	+
Elliot et al. [116]	1985	+	+	N/A	N/A	N/A	N/A	−	−	N/A
Fenemor et al. [106]	2022	+	+	+	+	N/A	N/A	+	−	+
Girard et al. [93]	2014	+	+	N/A	N/A	N/A	N/A	−	−	N/A
Girard et al. [92]	2014	+	+	N/A	N/A	N/A	N/A	−	−	N/A
Goodman et al. [99]	1985	+	+	−	N/A	N/A	N/A	−	−	+
Henderson et al. [30]	2020	+	+	+	+	N/A	−	+	+	N/A
Hornery et al. [112]	2007	+	+	N/A	+	−	N/A	−	+	N/A
Huang et al. [113]	2022	+	+	N/A	N/A	N/A	N/A	−	+	N/A
Knez and Périard [97]	2014	+	+	N/A	N/A	N/A	N/A	−	−	N/A
Kouassi et al. [87]	2019	+	−	N/A	N/A	N/A	−	−	+	N/A
Mohr et al. [88]	2004	+	−	−	N/A	N/A	N/A	−	−	N/A
Mohr et al. [119]	2012	−	+	+	−	+	N/A	−	−	+
Morante and Brotherhood [102]	2008	+	+	N/A	N/A	N/A	−	+	−	N/A
Morante and Brotherhood [100]	2008	−	−	N/A	N/A	N/A	−	+	−	−
Morante and Brotherhood [101]	2007	+	+	N/A	N/A	N/A	−	+	−	N/A
Naito et al. [114]	2022	+	+	N/A	−	−	−	+	+	N/A
Ozgunen et al. [111]	2010	+	+	−	+	+	N/A	−	−	+
Périard et al. [94]	2014	+	+	N/A	N/A	N/A	N/A	−	−	N/A
Périard et al. [95]	2014	+	+	N/A	N/A	N/A	N/A	−	−	N/A
Périard et al. [96]	2014	+	+	N/A	N/A	N/A	N/A	−	−	N/A
Pliauga et al. [89]	2015	+	−	+	N/A	N/A	N/A	−	−	N/A
Stay et al. [82]	2018	−	+	N/A	+	−	N/A	−	+	N/A
Taylor et al. [91]	2019	+	+	−	+	N/A	N/A	+	−	+
Tippet et al. [107]	2011	+	+	N/A	+	N/A	−	−	−	N/A
Veale and Pearce [115]	2009	+	+	+	+	N/A	N/A	−	−	N/A

**+ **satisfies methodological checklist element; − does not satisfy methodological checklist element; *N/A* not applicable

## Discussion

### Summary of Main Results

This systematic review aimed to synthesise the research findings regarding the *T*_c_ responses to intermittent sports competition. The majority of included studies identified magnitudes of *T*_c_ that have been associated with hyperthermia (and related performance and health effects) [28] occurring during intermittent sports competition across sports, competition levels, sexes, exposure times and environmental conditions. Our findings show that athletes, coaches, practitioners and/or policymakers are in many cases justified to seek methods that may limit the heat strain experienced by the competing athletes in intermittent sports such as heat acclimatisation/acclimation training interventions [74] and mixed-method cooling in and around competition [21]. However, there is variation in peak *T*_c_ among the studies included in this review, likely resulting from a complex interplay between the physical intensity and duration of the exposure, environmental conditions during competition, methods of measurement and athlete genetics (modulated by the magnitude of heat acclimatisation/acclimation). More detailed reporting of contextual data, as well as greater standardisation of reporting is necessary to gain a higher-resolution understanding of the relationships between these factors and peak *T*_c_ through meta-analytic statistical methods such as meta-regression. This would allow stronger inferences to be drawn, and athletes, coaches, practitioners and policymakers would be able to intervene more confidently to improve the performance and/or health of competing athletes (and minimise the likelihood of intervening negatively or unnecessarily).

### Methodological Considerations

Whilst the findings of the present review provide an important synthesis of the available data collected from intermittent sport athletes, we anticipated (based on prior scoping research and expert consultation) that, given the heterogeneity of the methods within the included studies, informative summary estimates and moderators could not be calculated. Nonetheless, the combined results show a common presence of hyperthermia (Fig. 4) of magnitudes shown to impair performance [28]. Most included studies reported either WBGT or ambient temperature and relative humidity and the exposure time in competition, and clearly described their population (particularly the studies from 2010 onwards). These parameters (environmental conditions, exposure time and population) are arguably the most significant contributors to heat strain in intermittent sport athletes. Investigating the impact of these variables on *T*_c_ (through statistical modelling techniques) will improve the ability to anticipate and mitigate potential heat strain in the future. This enables support staff and policymakers to optimise their interventions for performance and safety with greater precision. A further methodological improvement is the use of *T*_c_ measurement technology capable of continuous data monitoring (as opposed to capturing data at discrete time points during breaks in play). This allows a more valid assessment of peak *T*_c_, as the peak is likely to occur during play after prolonged, intense and metabolically demanding actions. Although this technology is not new (i.e. a sampling frequency of 60 s was used in earlier studies by Morante and Brotherhood in 2007–2008 [100–102]), improvements in technology and decreased cost in recent years have made acquiring such devices much more feasible for researchers and practitioners. Overall, the volume of studies identified by this systematic search and their combined results (interpreted within the context of methodological quality) indicate that the relationship between intermittent sports competition and high *T*_c_ is trustworthy, although the independent effects of WBGT and exposure time require further research and analysis before explanatory models with informative summary estimates and moderators can be developed.

### Limitations and Potential Biases in the Review Process

The primary limitation of the present review is the heterogeneity between studies preventing the combined data from being appropriately meta-analysed. More research is needed between sports (and associated competition durations), environmental conditions and levels of competition to attain sufficient homogeneity for informative statistical synthesis. A current gap in the body of evidence presented is the relatively low volume of research on athletes competing at the highest levels of intermittent sport (level 5 based on Russell et al. [103] classification system; 9 out of 34 included studies or ~ 26%), where small changes are considered important and the implications on performance are of greatest consequence. Without sufficient statistical power from a larger volume of relevant data, the small but practically important changes in physiology and performance for athletes at this level [121] will remain undetected. Opportunities to improve performance through practical and effective interventions for intermittent sport athletes may therefore be missed and impact competition and financial outcomes [122]. A further limitation to the validity of these findings is the limited volume of continuous *T*_c_ data included. Most studies in this review used a discrete point-in-time measurement of *T*_c_, concealing the temporal changes occurring between datapoints and preventing the accurate calculation of summary statistics such as mean *T*_c_. A potential bias in the body of evidence presented in this review lies in the volume of data reported across multiple studies. Whilst different hypotheses and distinct methodologies are presented, there remains the potential for researchers to mistake the data within these studies as novel (as it is often not explicitly stated), generating duplicate data points during synthesis and more heavily weighting the findings towards the characteristics of these data.

### Implications for Practice and Future Research

The studies included in this systematic review contain data with high external validity, as they were collected in competition (competitive, friendly or experimental) with real external influencing factors (e.g. unpredictable opponents/defenders, specific technical skill execution, tactical decision-making, match/competition physical demands). Whilst this allows the findings to be highly generalisable, it comes at the expense of the high degree of experimental control possible in laboratory studies that can test hypotheses whilst tightly controlling for many of the external factors present in the real world. The best original research evidence derives from controlled trials where changes in a dependent variable are observed when altering one or many independent variables, but for transferability to occur from scientific research into practice, research from the field is required [123]. This enables researchers and practitioners to build upon the knowledge gained from controlled studies by practically applying the theory more broadly to determine efficacy and feasibility in the real world. While this systematic review is the first to examine heat strain during competition among intermittent sport athletes, the substantial body of research findings from endurance sports in comparable environmental conditions, combined with the preliminary results of this review, suggest that intermittent sport athletes frequently experience exercise-induced hyperthermia during competition. With the known performance and health effects of these magnitudes of hyperthermia, future research should investigate feasible practice-focused interventions for intermittent sport athletes. Strong scientific and applied support exists for heat acclimatisation/acclimation training interventions [27] and acute cooling interventions [124] based on research and practice from endurance athletes and practitioners. More research investigating the efficacy and practicality of these methods in intermittent sport athletes and settings is required before these findings can be universally considered best-practice management of high thermal strain during competition across all sports.

## Conclusion

This systematic review has synthesised the available thermal strain data collected in competition from intermittent sport athletes. Almost 90% of the studies that met the eligibility criteria found some degree of hyperthermia (*T*_c_ > 38.5 °C), with almost 20% of the studies finding ‘marked’ hyperthermia (*T*_c_ > 39.5 °C). Exercise-induced hyperthermia has been associated with a range of negative performance and health outcomes in athletes. Practitioners and coaches supporting intermittent sport athletes are justified to seek interventions aimed at mitigating the high heat strain observed in competition. More research is required to determine the most effective interventions that are practically viable in intermittent sports settings (often constrained by many competing demands). Greater statistical power and homogeneity among studies is required to quantify the independent effects of WBGT, competition duration, sport and level of competition on peak *T*_c_, all likely to be key modulators of the thermal strain experienced by competing athletes.

### Supplementary Information

Below is the link to the electronic supplementary material.

Supplementary file1 (PDF 244 KB)

Supplementary file2 (DOCX 15 KB)

Supplementary file3 (XLSX 197 KB)

Supplementary file4 (DOCX 950 KB)

Supplementary file5 (DOCX 52 KB)

Supplementary file6 (DOCX 48 KB)

## Declarations

### Data Availability

All data generated or analysed during this study are included in this published article (and its supplementary information files).
