# Supplementary material for: Core Body Temperatures in Intermittent Sports: A Systematic Review
Source: Sports Med. 2023 Aug 1;53(11):2147–70. doi: 10.1007/s40279-023-01892-3 (PMC10587327; doi:10.1007/s40279-023-01892-3)
Supplement: Supplementary file 1 — Supplementary file1 (PDF 244 KB) [file 40279_2023_1892_MOESM1_ESM.pdf]

Search strategy for

Core body temperature in intermittent sports: A systematic review

using {litsearchr}

Mitch Henderson

07/01/2021

The full code (without explanation text) is available here in the OSF repository for this review.

A large portion of the code explanations in this document have been taken from Luke Tudge's tutorial of `litsearchr`.

Introduction

Partially automating evidence synthesis reduces the amount of human time and effort needed to conduct a systematic review and reduce bias in keyword selection when researchers develop search strategies. The `litsearchr` R package facilitates quick, objective, reproducible search strategy development using text-mining and keyword co-occurrence networks to identify important terms to include in a search strategy as described in Grames et al. (2019). It can automatically write Boolean search strings with stemming support. To assess the quality of a search, it can also check the results of a search against a set of known, relevant articles to get performance metrics. This report will document how I went through this process for my systematic review titled "Core body temperatures in intermittent sports: A systematic review".

Install `litserachr`

As `litsearchr` is not currently on CRAN, it can be downloaded from Github using `remotes::install_github("elizagrames/litsearchr", ref="main")`.

```
library(tidyverse)
library(litsearchr)
library(igraph)
library(here)
```

`litsearchr` is a new package and is currently in development. So we should keep track of which version we are using, in case we later work with a newer version and find that the this script no longer works.

```r
packageVersion("litsearchr")
```

```
## [1] '1.0.0'
```

Write and conduct naive search

The naive search was `("intermittent sport*" OR "team sport*" OR athlete* OR player*) AND ("core temperature*" OR "body temperature*")`. This resulted in:

Ovid MEDLINE returning 800 records

Web of Science returning 642 records

SPORTDiscus returning 940 records

```r
naiveimport <- import_results(
  directory = here("naive_results"),
  verbose = TRUE)
```

```
## Reading file C:/Users/Mitch.Henderson/OneDrive - UTS/Research/PhD/Tc Team
Sport Systematic Review/naive_results/naive_search_Ovid_MEDLINE.ris ... done
## Reading file C:/Users/Mitch.Henderson/OneDrive - UTS/Research/PhD/Tc Team
Sport Systematic Review/naive_results/naive_search_SportDISCUS.txt ... done
## Reading file C:/Users/Mitch.Henderson/OneDrive - UTS/Research/PhD/Tc Team
Sport Systematic Review/naive_results/naive_search_WOS_1-500.bib ... done
## Reading file C:/Users/Mitch.Henderson/OneDrive - UTS/Research/PhD/Tc Team
Sport Systematic Review/naive_results/naive_search_WOS_501-642.bib ... done
```

```r
naiveresults <- naiveimport %>%
  remove_duplicates(field = "title", method = "string_osa")
```

2,379 records in total from the 3 databases.

1,527 after duplicates removed.

Establish stopwords

Some of the phrases that appear are general science or data analysis terms and are not related to the specific topic of the article. In language analysis, such frequently-occurring but uninformative words are often called 'stopwords'. I have prepared a (non-exhaustive) list of generic sport science research words that occurred in this set of articles.

```r
sport_science_stopwords <- c("significant",
                             "significantly",
                             "results",
                             "findings",
                             "rights",
                             "outcome",
                             "study",
                             "effect",
```

```
                              "control",
                              "controlled",
                              "confidence",
                              "measure",
                              "measures")
```

The `extract_terms()` function (that I'll use below to identify the key terms) provides a stopwords argument that we can use to filter out words. `litsearchr` also provides a general list of stopwords for English (and for some other languages) via the get_stopwords() function, so we I'll add this to my more specific stopwords.

```
all_stopwords <- c(get_stopwords("English"), sport_science_stopwords)
```

```
## Loading required namespace: stopwords
```

Extract keywords

This will look through the articles from my imported bibliographic files and use text mining to detect common terms from the titles, abstracts, and keywords.

```
rakedkeywords <-
  extract_terms(
    text = paste(naiveresults$title, naiveresults$abstract),
    method = "fakerake",
    min_freq = 2,
    ngrams = TRUE,
    min_n = 2,
    language = "English",
    stopwords = all_stopwords
  )


taggedkeywords <-
  extract_terms(
    keywords = naiveresults$keywords,
    method = "tagged",
    min_freq = 2,
    ngrams = TRUE,
    min_n = 2,
    language = "English",
    stopwords = all_stopwords
  )
```

The min_freq = 2 argument means only keywords that appear at least twice in the full set of results will be returned. This is good for making sure that we are only getting keywords that are related to more than just one article in our field of interest.

The min_n = 2 argument means only get keywords that consist of at least two words. This is why we only see multi-word phrases returned within these objects. As the `rakedkeywords` object below results in over 5,000 terms, reducing this `min_n` to 1 would be too broad and return too many irrelevant results.

Build the keyword co-occurrence network

Now we have our list of identified search terms, but there are probably still some in there that are unrelated to the others and to our topic of interest. These perhaps only occur in a small number of articles that do not mention many of the other search terms. We would like some systematic way of identifying these 'isolated' search terms.

One way to do this is to analyze the search terms as a network. The idea behind this is that terms are linked to each other by virtue of appearing in the same articles. If we can find out which terms tend to occur together in the same article, we can pick out groups of terms that are probably all referring to the same topic (because they occur in the same subset of articles), and we can filter out terms that do not often occur together with any of the main groups of terms.

This section of code combines the keywords, creates a document feature matrix, and then a network of that matrix.

```
all_keywords <- unique(append(taggedkeywords, rakedkeywords))

naivedfm <-
  create_dfm(
    elements = paste(naiveresults$title, naiveresults$abstract),
    features = all_keywords
  )

naivegraph <-
  create_network(
    search_dfm = as.matrix(naivedfm),
    min_studies = 2,
    min_occ = 2
  )
```

Identify change points in keyword importance

The strength of the network can now be used to rank search terms by importance, with the aim of pruning away some of the least important ones. That's what the `find_cutoff()` function does.

The `cumulative` method selected captures the chosen top percentage of frequently occurring terms.

```
strengths <- strength(naivegraph)

term_strengths <- data.frame(term = names(strengths),
                             strength = strengths,
                             row.names = NULL) %>%
 mutate(rank = rank(strength, ties.method = "min")) %>%
 arrange(strength)

cutoff <-
  find_cutoff(
    naivegraph,
    method = "cumulative",
    percent = .20,
    imp_method = "strength"
  )
```

```
ggplot(term_strengths, aes(x=rank, y=strength, label=term)) +
 geom_line() +
 geom_point() +
 geom_text(data = filter(term_strengths, rank > 5),
           hjust = "right",
           nudge_y = 20,
           check_overlap = TRUE) +
 geom_hline(yintercept = cutoff, linetype = "dashed")
```

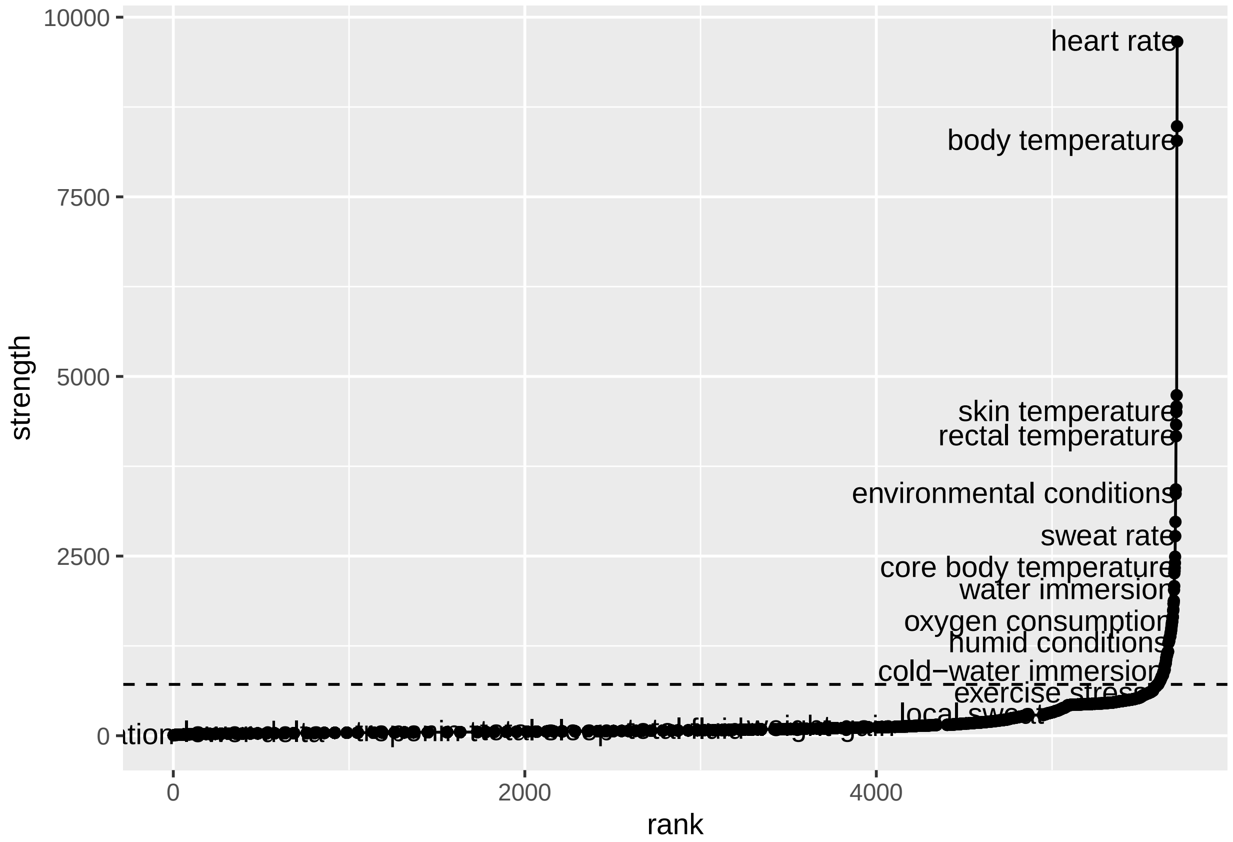

Based on visual inspection of the above line plot, the top 20% was chosen as an appropriate cutoff (this represented a rough infliction point of the frequencies).

Create reduced network

This section applies our chosen cutoff to create a 'reduced' network and then extract the keywords.

```
reducedgraph <-
  reduce_graph(naivegraph, cutoff_strength = cutoff[1])

searchterms <- get_keywords(reducedgraph)
```

Add relevent one word terms

As we only requested a minimum of 2 or more word phrases when extracting terms with `extract_terms()` (to prevent excess irrelevant results), we can manually add in any one word terms that we know are relevant and combine them with our established search terms.

```
extra_terms <- c(
  "players", "match-play", "matchplay", "match play",
  "competition", "cricket", "football", "basketball",
  "netball", "hockey", "lacrosse", "softball",
  "rugby", "volleyball", "baseball", "soccer",
  "handball", "tennis", "boxing", "athletes",
  "polo", "boxers"
)

selected_terms <- sort(c(searchterms, extra_terms))
```

Group terms into concepts

This step is done manually so `litsearchr` can create the search string using `OR` to connect terms *within* the groups, and `AND` to connect the groups. I used the PEO (population, exposure, outcome) framework to guide my groups. Below, I've manually provided each term from my `selected_terms` object to either no group (identified term does not fit into any group), one group (e.g. "core temperature" as an outcome), or multiple groups (e.g. football as a population term [football players, footballers] or an exposure term ["…in professional football matches"]).

```
grouped_terms <-list(
  population = selected_terms[c(3,4,6,7,17,29,33,46,47,50,51,52,54,61,69,79,90,92,98,
                                101,102,103,104,113,115,123,124,127,130,131)],
  exposure = selected_terms[c(3,5,6,7,18,23,29,50,54,61,69,71,72,73,79,92,98,101,104,
                              106,113,115,127)],
  outcome = selected_terms[c(15,27,28,96)]
)

grouped_terms
```

```
## $population
##  [1] "american football" "athletes"          "baseball"
##  [4] "basketball"        "boxers"            "cricket"
##  [7] "elite athlete"     "female athlete"    "female athletes"
## [10] "football"          "football player"   "football players"
## [13] "handball"          "hockey"            "lacrosse"
## [16] "netball"           "players"           "polo"
## [19] "rugby"             "soccer"            "soccer player"
## [22] "soccer players"    "softball"          "team sport"
## [25] "tennis"            "trained athlete"   "trained athletes"
## [28] "volleyball"        "young athlete"     "young athletes"
##
## $exposure
##  [1] "american football"    "athletic performance" "baseball"
##  [4] "basketball"           "boxing"               "competition"
```

```
##  [7] "cricket"             "football"            "handball"
## [10] "hockey"              "lacrosse"            "match-play"
## [13] "match play"          "matchplay"           "netball"
## [16] "polo"                "rugby"               "soccer"
## [19] "softball"            "sports performance"  "team sport"
## [22] "tennis"              "volleyball"
##
## $outcome
## [1] "body temperature"      "core body temperature" "core temperature"
## [4] "rectal temperature"
```

Write Boolean searches

The `write_search()` function takes our list of grouped search terms and writes the text of a new search.

```
my_search <-
  write_search(
    groupdata = grouped_terms,
    languages = c("English"),
    stemming = TRUE,
    closure = "none",
    exactphrase = TRUE,
    writesearch = F,
    verbose = TRUE
  )
```

```
## [1] "English is written"
```

```
my_search
```

```
## [1] "((athlet* OR basebal* OR basketbal* OR boxer* OR cricket* OR \"elit*
athlet*\" OR \"femal* athlet*\" OR footbal* OR handbal* OR hockey* OR lacross*
OR netbal* OR player* OR polo* OR rugbi* OR soccer* OR softbal* OR \"team*
sport*\" OR tenni* OR \"train* athlet*\" OR volleybal* OR \"young* athlet*\")
AND (\"athlet* perform*\" OR basebal* OR basketbal* OR boxing OR competit* OR
cricket* OR footbal* OR handbal* OR hockey* OR lacross* OR match-play* OR
\"match* play*\" OR matchplay* OR netbal* OR polo* OR rugbi* OR soccer* OR
softbal* OR \"sport* perform*\" OR \"team* sport*\" OR tenni* OR volleybal*)
AND (\"bodi* temperatur*\" OR \"core* temperatur*\" OR \"rectal*
temperatur*\"))"
```

The stemming algorithm used to create this search has some imperfections and the creator of the `litsearchr` package (Eliza Grames from the University of Connecticut) has recommended to me in personal discussion to use a text editor to manually remove:

1. the letter 'i' at the end of words that previously had 'y' (this is the algorithm trying to account for multiple suffixes such as society and societies)
2. the backslash character present before quotation marks (this is an escape character for R)

I've also manually removed any redundant terms that are encapsulated by other terms (for example "elite athlete" when "athlete" is present elsewhere).

After making these adjustments (except keeping the backslashes needed to print in R for this example), the final search was:

```
read_lines(here("search-inEnglish_clean.txt"))
```

```
## [1] "((athlet* OR basebal* OR basketbal* OR boxer* OR cricket* OR footbal*
OR handbal* OR hockey* OR lacross* OR netbal* OR player* OR polo* OR rugb* OR
soccer* OR softbal* OR \"team* sport*\" OR tenni* OR volleybal*) AND (\"athlet*
perform*\" OR basebal* OR basketbal* OR boxing OR competit* OR cricket* OR
footbal* OR handbal* OR hockey* OR lacross* OR match-play* OR \"match* play*\"
OR matchplay* OR netbal* OR polo* OR rugb* OR soccer* OR softbal* OR \"sport*
perform*\" OR \"team* sport*\" OR tenni* OR volleybal*) AND (\"bod*
temperatur*\" OR \"core* temperatur*\" OR \"rectal* temperatur*\"))"
```

This search resulted in:

Ovid MEDLINE returning 868

Web of Science returning 621

SPORTDiscus returning 624

Check search strategy precision and recall

To do this, I first create a vector of the title names of articles that I know are relevant for my review.

```
gold_standard <-
  c("Greater chance of high core temperatures with modified pacing strategy during team sport in the hea
    "Core body temperature during competition in the heat: national boys' 14s junior tennis championshi
    "Core temperature responses and match running performance during intermittent-sprint exercise compe
    "Thermoregulatory observations in soccer match play: professional and recreational level applicatio
    "Changes in Core Temperature During an Elite Female Rugby Sevens Tournament",
    "An integrated physiological and performance profile of professional tennis",
    "Physiological Responses and Physical Performance during Football in the Heat",
    "Air temperature and physiological and subjective responses during competitive singles tennis",
    "Autonomic and behavioural thermoregulation in tennis",
    "Effect of hot environmental conditions on physical activity patterns and temperature response of f
    "Thermal, physiological and perceptual strain mediate alterations in match-play tennis under heat s
    "Core Temperature Responses in Elite Cricket Players during Australian Summer Conditions",
    "Alterations in core temperature during World Rugby Sevens Series tournaments in temperate and warm
    "Core Temperature and Sweat Responses in Professional Women's Tennis Players During Tournament Play
  )

title_search <- write_title_search(titles = gold_standard)
```

Then I'll import my bibliographic files that the full search returned, and remove duplicates.

```
retrieved_articles <-
  import_results(
    directory = here("full_search"),
    verbose = TRUE)
```

```
## Reading file C:/Users/Mitch.Henderson/OneDrive - UTS/Research/PhD/Tc Team
Sport Systematic Review/full_search/full_search_OvidMEDLINE.ris ... done
## Reading file C:/Users/Mitch.Henderson/OneDrive - UTS/Research/PhD/Tc Team
Sport Systematic Review/full_search/full_search_SportDISCUS.txt ... done
## Reading file C:/Users/Mitch.Henderson/OneDrive - UTS/Research/PhD/Tc Team
Sport Systematic Review/full_search/full_search_WOS_1-500.bib ... done
## Reading file C:/Users/Mitch.Henderson/OneDrive - UTS/Research/PhD/Tc Team
Sport Systematic Review/full_search/full_search_WOS_501-621.bib ... done
```

```
retrieved_articles <- remove_duplicates(retrieved_articles,
                                        field = "title",
                                        method = "string_osa")
```

2,111 articles were retrieved in total (2 import errors).

1,390 after duplicates were removed.

Now, I compare the full collection of retrieved articles with the titles I listed above to see that they were all found.

```
articles_found <- data.frame(check_recall(true_hits = gold_standard,
                                          retrieved = retrieved_articles$title))

articles_found
```

```
## Title
## 1 Greater chance of high core temperatures with modified pacing strategy
during team sport in the heat
## 2 Core body temperature during competition in the heat: national boys' 14s
junior tennis championships
## 3 Core temperature responses and match running performance during
intermittent-sprint exercise competition in warm conditions
## 4 Thermoregulatory observations in soccer match play: professional and
recreational level applications using an intestinal pill system to measure core
temperature
## 5 Changes in Core Temperature During an Elite Female Rugby Sevens Tournament
## 6 An integrated physiological and performance profile of professional tennis
## 7 Physiological Responses and Physical Performance during Football in the
Heat
## 8 Air temperature and physiological and subjective responses during
competitive singles tennis
## 9 Autonomic and behavioural thermoregulation in tennis
## 10 Effect of hot environmental conditions on physical activity patterns and
temperature response of football players
## 11 Thermal, physiological and perceptual strain mediate alterations in
match-play tennis under heat stress
## 12 Core Temperature Responses in Elite Cricket Players during Australian
Summer Conditions
## 13 Alterations in core temperature during World Rugby Sevens Series
tournaments in temperate and warm environments
## 14 Core Temperature and Sweat Responses in Professional Women's Tennis
Players During Tournament Play in the Heat
## Best_Match
## 1 Greater chance of high core temperatures with modified pacing strategy
```

```
during team sport in the heat
## 2 Core body temperature during competition in the heat: National Boys' 14s
Junior Championships
## 3 Core temperature responses and match running performance during
intermittent-sprint exercise competition in warm conditions
## 4 Thermoregulatory observations in soccer match play: professional and
recreational level applications using an intestinal pill system to measure core
temperature
## 5 Changes in Core Temperature During an Elite Female Rugby Sevens Tournament
## 6 An integrated physiological and performance profile of professional tennis
## 7 Physiological responses and physical performance during football in the
heat
## 8 Air temperature and physiological and subjective responses during
competitive singles tennis
## 9 Autonomic and behavioural thermoregulation in tennis
## 10 Effect of hot environmental conditions on physical activity patterns and
temperature response of football players
## 11 Thermal, physiological and perceptual strain mediate alterations in
match-play tennis under heat stress
## 12 Core Temperature Responses in Elite Cricket Players during Australian
Summer Conditions
## 13 Alterations in core temperature during World Rugby Sevens Series
tournaments in temperate and warm environmentsâ\200
## 14 Core temperature and sweat responses in professional women's tennis
players during tournament play in the heat
## Similarity
## 1 1
## 2 0.715789473684211
## 3 1
## 4 1
## 5 1
## 6 1
## 7 1
## 8 1
## 9 1
## 10 1
## 11 1
## 12 1
## 13 0.986666666666667
## 14 1
```

All articles were found in the search, providing confidence that this search is appropriate.

```
sessionInfo()
```

```
## R version 4.0.3 (2020-10-10)
## Platform: x86_64-w64-mingw32/x64 (64-bit)
## Running under: Windows 10 x64 (build 19042)
##
## Matrix products: default
##
## locale:
## [1] LC_COLLATE=English_Australia.1252  LC_CTYPE=English_Australia.1252
```

```
## [3] LC_MONETARY=English_Australia.1252 LC_NUMERIC=C
## [5] LC_TIME=English_Australia.1252
##
## attached base packages:
## [1] stats     graphics  grDevices utils     datasets  methods   base
##
## other attached packages:
##  [1] here_1.0.1       igraph_1.2.6     litsearchr_1.0.0 forcats_0.5.1
##  [5] stringr_1.4.0    dplyr_1.0.5      purrr_0.3.4      readr_1.4.0
##  [9] tidyr_1.1.3      tibble_3.1.1     ggplot2_3.3.3    tidyverse_1.3.1
## [13] knitr_1.32
##
## loaded via a namespace (and not attached):
##  [1] Rcpp_1.0.7         stringdist_0.9.6.3 lubridate_1.7.10   assertthat_0.2.1
##  [5] rprojroot_2.0.2    digest_0.6.27      utf8_1.1.4         slam_0.1-48
##  [9] R6_2.5.0           cellranger_1.1.0   backports_1.2.0    reprex_2.0.0
## [13] evaluate_0.14      highr_0.8          httr_1.4.2         pillar_1.6.0
## [17] rlang_0.4.10       readxl_1.3.1       rstudioapi_0.13    rmarkdown_2.7
## [21] labeling_0.4.2     munsell_0.5.0      broom_0.7.6        compiler_4.0.3
## [25] modelr_0.1.8       xfun_0.22          pkgconfig_2.0.3    htmltools_0.5.1.1
## [29] tidyselect_1.1.0   fansi_0.4.1        crayon_1.4.1       dbplyr_2.1.1
## [33] withr_2.4.1        SnowballC_0.7.0    grid_4.0.3         jsonlite_1.7.2
## [37] gtable_0.3.0       lifecycle_1.0.0    DBI_1.1.1          magrittr_2.0.1
## [41] synthesisr_0.3.0   scales_1.1.1       cli_2.4.0          stringi_1.5.3
## [45] farver_2.0.3       fs_1.5.0           NLP_0.2-1          xml2_1.3.2
## [49] ellipsis_0.3.1     stopwords_2.1      generics_0.1.0     vctrs_0.3.7
## [53] tools_4.0.3        glue_1.4.2         hms_1.0.0          parallel_4.0.3
## [57] yaml_2.2.1         tm_0.7-8           colorspace_2.0-0   rvest_1.0.0
## [61] ngram_3.0.4        haven_2.3.1        usethis_2.0.0
```
