# Supplementary material for: Core Body Temperatures in Intermittent Sports: A Systematic Review
Source: Sports Med. 2023 Aug 1;53(11):2147–70. doi: 10.1007/s40279-023-01892-3 (PMC10587327; doi:10.1007/s40279-023-01892-3)
Supplement: Supplementary file 2 — Supplementary file2 (DOCX 15 KB) [file 40279_2023_1892_MOESM2_ESM.docx]

**Supplementary material 2.** Boolean search strings and results for each database searched.

| **Database** | **Boolean search string** | **Limits** | **Results** |
| --- | --- | --- | --- |
|  |  |  |  |
| **Web of Science Core Collection** | **TOPIC:** ((athlet* *OR* basebal* *OR* basketbal* *OR* boxer* *OR* cricket* *OR* footbal* *OR* handbal* *OR* hockey* *OR* lacross* *OR* netbal* *OR* player* *OR* polo* *OR* rugb* *OR* soccer* *OR* softbal* *OR* "team* sport*" *OR* tenni* *OR* volleybal*) *AND* ("athlet* perform*" *OR* basebal* *OR* basketbal* *OR* boxing *OR* competit* *OR* cricket* *OR* footbal* *OR* handbal* *OR* hockey* *OR* lacross* *OR* match-play* *OR* "match* play*" *OR* matchplay* *OR* netbal* *OR* polo* *OR* rugb* *OR* soccer* *OR* softbal* *OR* "sport* perform*" *OR* "team* sport*" *OR* tenni* *OR* volleybal*) *AND* ("bod* temperatur*" *OR* "core* temperatur*" *OR* "rectal* temperatur*")) | N/A | 760 |
| **Ovid MEDLINE** | ((athlet* or basebal* or basketbal* or boxer* or cricket* or footbal* or handbal* or hockey* or lacross* or netbal* or player* or polo* or rugb* or soccer* or softbal* or "team* sport*" or tenni* or volleybal*) and ("athlet* perform*" or basebal* or basketbal* or boxing or competit* or cricket* or footbal* or handbal* or hockey* or lacross* or match-play* or "match* play*" or matchplay* or netbal* or polo* or rugb* or soccer* or softbal* or "sport* perform*" or "team* sport*" or tenni* or volleybal*) and ("bod* temperatur*" or "core* temperatur*" or "rectal* temperatur*")) | N/A | 998 |
| **EBSCOhost SPORTDiscus with Full Text** | ((athlet* OR basebal* OR basketbal* OR boxer* OR cricket* OR footbal* OR handbal* OR hockey* OR lacross* OR netbal* OR player* OR polo* OR rugb* OR soccer* OR softbal* OR "team* sport*" OR tenni* OR volleybal*) AND ("athlet* perform*" OR basebal* OR basketbal* OR boxing OR competit* OR cricket* OR footbal* OR handbal* OR hockey* OR lacross* OR match-play* OR "match* play*" OR matchplay* OR netbal* OR polo* OR rugb* OR soccer* OR softbal* OR "sport* perform*" OR "team* sport*" OR tenni* OR volleybal*) AND ("bod* temperatur*" OR "core* temperatur*" OR "rectal* temperatur*")) | N/A | 704 |
