# Supplementary material for: Core Body Temperatures in Intermittent Sports: A Systematic Review
Source: Sports Med. 2023 Aug 1;53(11):2147–70. doi: 10.1007/s40279-023-01892-3 (PMC10587327; doi:10.1007/s40279-023-01892-3)
Supplement: Supplementary file 4 — Supplementary file4 (DOCX 950 KB) [file 40279_2023_1892_MOESM4_ESM.docx]

**Supplementary material 4A.** Meta-analysis of eligible studies.

This meta-analysis synthesised the 35 eligible estimates reported in 26 studies and included 1,198 observations. The pooled mean based on the three-level random effects meta-analytic model of untransformed (raw) means was 39.0°C (95% CI: 38.8 – 39.2). Effects were pooled using the inverse variance method and τ^2^ was estimated using restricted maximum-likelihood. The prediction interval based on the *t*-distribution (degrees of freedom = 33) was 37.8 – 40.1°C. Prediction intervals provide an estimate of the range in which the true effect of an exposure is likely to lie in future studies. The proportion of total variation in mean estimates due to true between-study heterogeneity rather than chance or sampling error (after accounting for within-study heterogeneity) was very high (*I^2^* = 97%). The estimated value of τ² was 0.303 (0.1727 from between study variance and 0.1302 from within studies), indicating high heterogeneity among the effect sizes. Cochran’s Q test results (*p* < 0.01) indicate that the observed heterogeneity among the studies is unlikely to have occurred by chance alone. Medians and interquartile ranges reported in Stay et al. [1] were transformed to estimated means and standard deviations using the method outlined in Luo et al. [2]. Data from Blanksby et al. [3] was excluded due to not reporting any measure of variability in core body temperature. All analyses included in supplementary material 4 were performed using R statistical software [4] and the {meta} package [5].


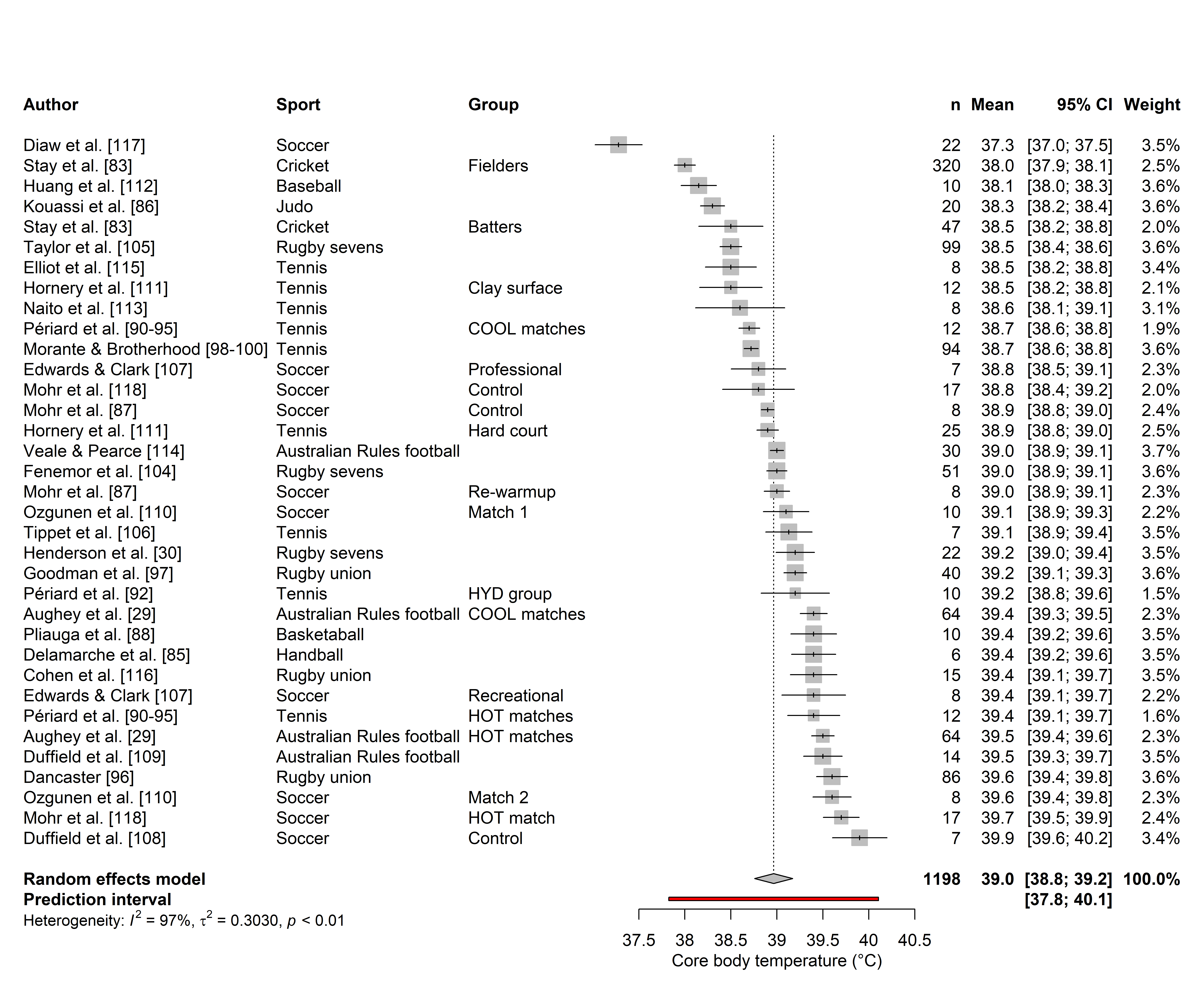


**Supplementary material 4B.** Subgroup-analysis of eligible studies with exposure times of greater or less than 85 minutes

This subgroup analysis was conducted to explore potential heterogeneity related to variation in exposure time. Subgroups were defined by involvement durations of greater or less than 85 minutes. The subgroup including exposures greater than 85 minutes included 17 estimates and 315 participants, whereas the subgroup including exposures less than 85 minutes included 14 studies and 815 participants. The pooled mean estimate was 39.0°C (95% CI: 38.7 – 39.3) for both subgroups. Tests for subgroup differences were not statistically significant (*p* = 0.77). Data from Blanksby et al. [3] was excluded due to not reporting any measure of variability in core body temperature. Data from Stay et al. [1] and Mohr et al. [6] were excluded due to not reporting an absolute measure of exposure duration.


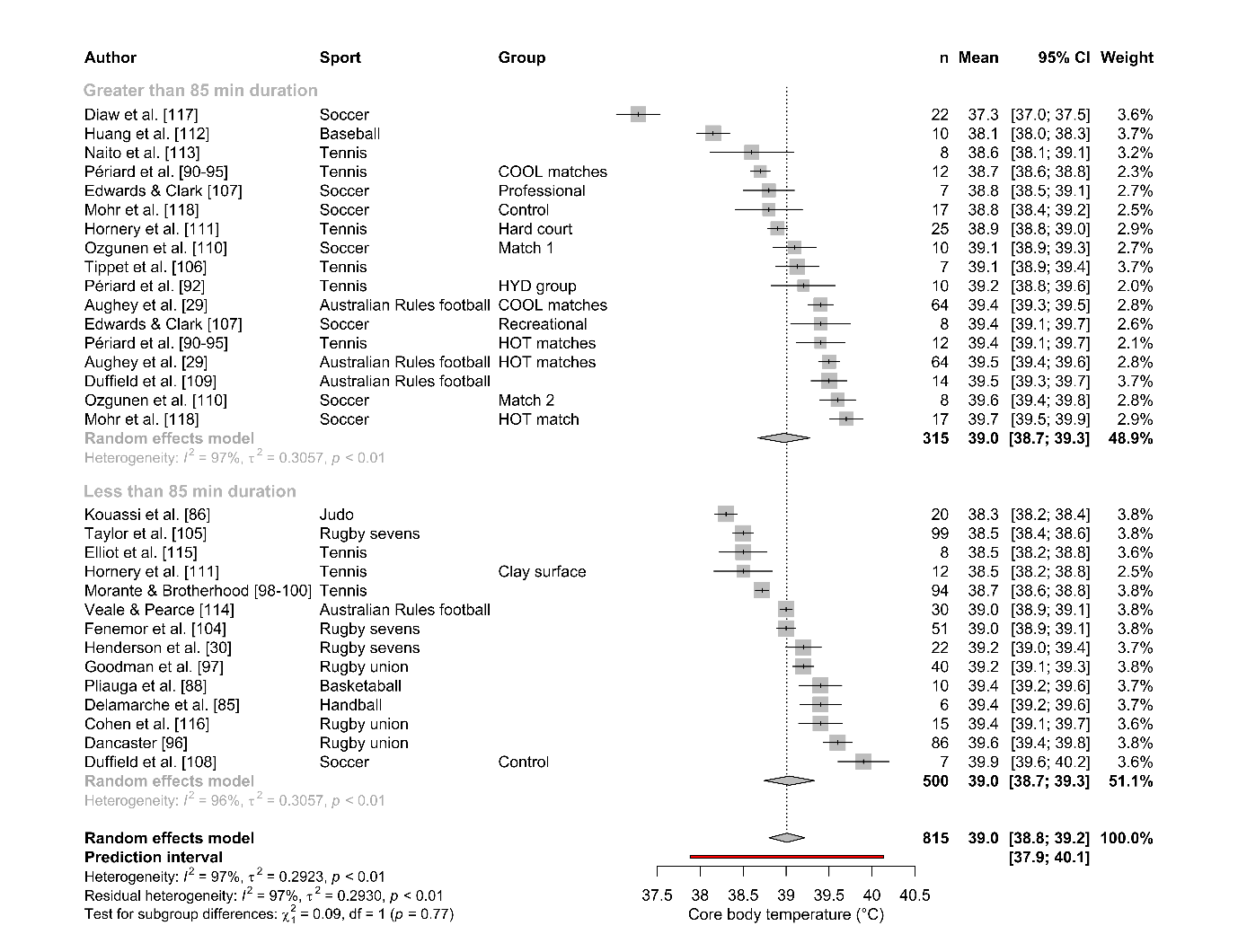


**Supplementary material 4C.** Subgroup-analysis of eligible studies with wet bulb globe temperatures of above or below 24°C

This subgroup analysis was conducted to explore potential heterogeneity related to variation in wet bulb globe temperature (WBGT). Subgroups were defined by WBGT values above or below 24°C. The subgroup including WBGT values above 24°C included 15 estimates and 593 participants, whereas the subgroup including WBGT values below 24°C included 15 studies and 553 participants. The pooled mean estimate was 39.1°C (95% CI: 38.8 – 39.4) for the subgroup including WBGT values above 24°C, and 38.8°C (95% CI: 38.5 – 39.1) for the subgroup including WBGT values below 24°C. Tests for subgroup differences were not statistically significant (*p* = 0.10). When WBGT was not reported in text, estimates were calculated using the validated Liljegren method [7]. In the case of a maximum WBGT threshold being reported (e.g., < 18°C), the upper limit was used. In the case of a WBGT range being reported (min-max), the midpoint of the range was used. Medians and interquartile ranges for core body temperature reported in Stay et al. [1] were transformed to estimated means and standard deviations using the method outlined in Luo et al. [2]. Data from Delamarche et al. [8], Kouassi et al. [9], Mohr et al. [6], and Pliauga et al. [10] were excluded due to insufficient environmental data being reported to calculate a WBGT estimate. Data from Blanksby et al. [3] was excluded due to not reporting any measure of variability in core body temperature.


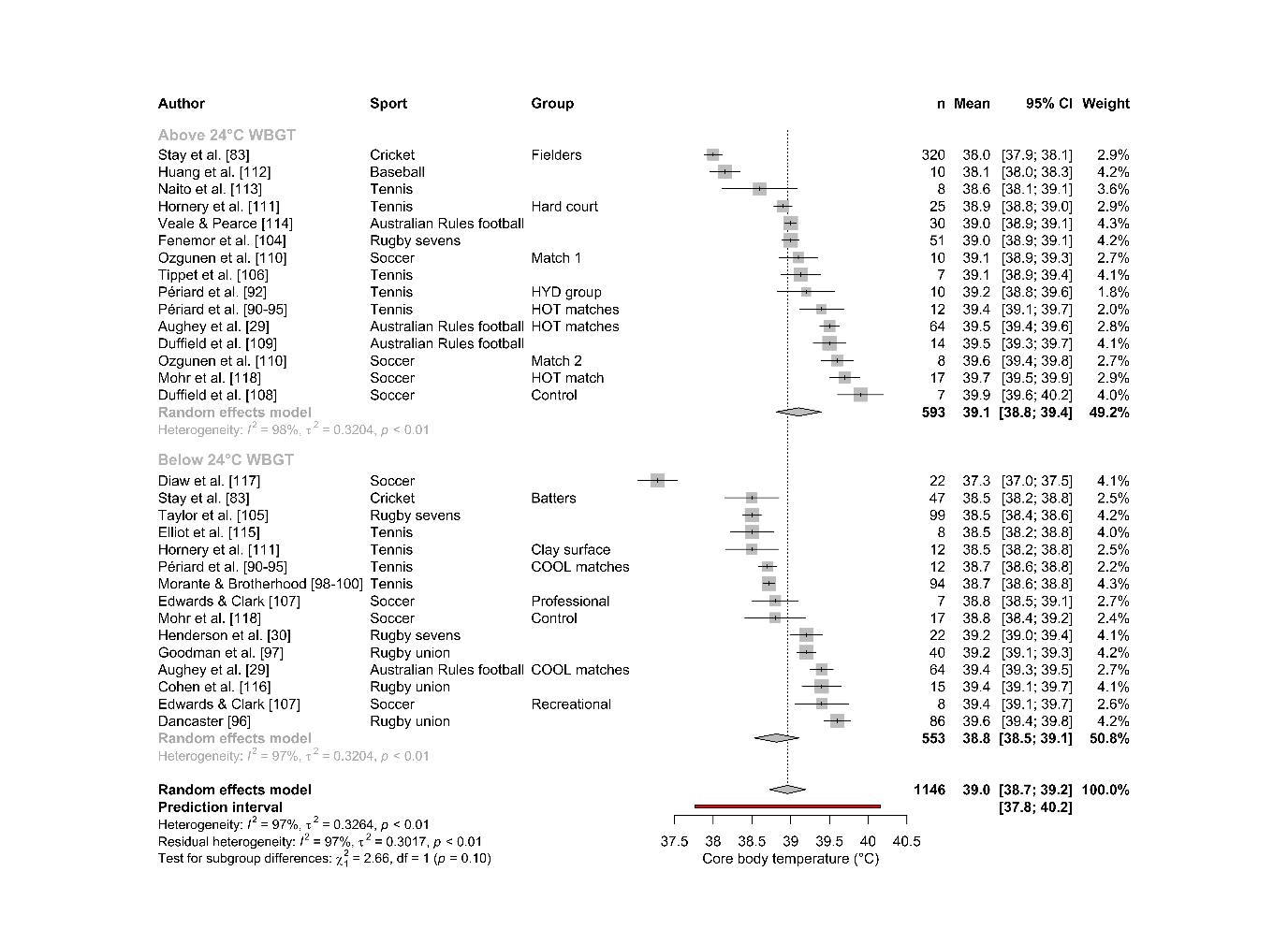
 **Reference list**

1. Stay S, Cort M, Ward D, Kountouris A, Orchard J, Holland J, et al. Core Temperature Responses in Elite Cricket Players during Australian Summer Conditions. Sports (Basel). 2018;6(4).

2. Luo D, Wan X, Liu J, Tong T. Optimally estimating the sample mean from the sample size, median, mid-range, and/or mid-quartile range. Stat Methods Med Res. 2018;27(6):1785-805.

3. Blanksby BA, Elliott BC, Davis KH, Mercer MD. Blood pressure and rectal temperature responses of middle-aged sedentary, middle-aged active and "A"-grade competitive male squash players. Br J Sports Med. 1980;14(2/3):133-8.

4. R Core Team. R: A language and environment for statistical computing. R Foundation for Statistical Computing, Vienna, Austria. 2022: <https://www.R-project.org/>.

5. Balduzzi S, Rucker G, Schwarzer G. How to perform a meta-analysis with R: a practical tutorial. Evid Based Ment Health. 2019;22:153-60.

6. Mohr M, Krustrup P, Nybo L, Nielsen JJ, Bangsbo J. Muscle temperature and sprint performance during soccer matches – beneficial effect of re-warm-up at half-time. Scand J Med Sci Sports. 2004;14(3):156-62.

7. Liljegren JC, Carhart RA, Lawday P, Tschopp S, Sharp R. Modeling the Wet Bulb Globe Temperature Using Standard Meteorological Measurements. J Occup Environ Hyg. 2008;5(10):645-55.

8. Delamarche P, Gratas A, Beillot J, Dassonville J, Rochcongar P, Lessard Y. Extent of lactic anaerobic metabolism in handballers. Int J Sports Med. 1987;8(1):55-9.

9. Kouassi J-P, Kouamé NG, Gouthon P, Gouthon G, Bio-Nigan I, Coulibaly S, et al. Effect of rapid voluntary weight loss on anthropometric, physiological and hydroelectrolytic parameters in elite judokas on the day of the 2018 Côte d'Ivoire National Championship. Biol Exerc. 2019;15(2):69-81.

10. Pliauga V, Kamandulis S, Dargeviciute G, Jaszczanin J, Kliziene I, Stanislovaitiene J, et al. The Effect of a Simulated Basketball Game on Players' Sprint and Jump Performance, Temperature and Muscle Damage. J Hum Kinet. 2015;46(1):167-75.
