# Supplementary material for: Core Body Temperatures in Intermittent Sports: A Systematic Review
Source: Sports Med. 2023 Aug 1;53(11):2147–70. doi: 10.1007/s40279-023-01892-3 (PMC10587327; doi:10.1007/s40279-023-01892-3)
Supplement: Supplementary file 5 — Supplementary file5 (DOCX 52 KB) [file 40279_2023_1892_MOESM5_ESM.docx]

**Supplementary material 4.** List of excluded studies (n = 35)

| **Exclusion criteria** | **Reference** |
| --- | --- |
|  |  |
| **No match data (n = 21)** | Chalmers et al. [1], Crowther et al. [2], Driscoll et al. [3], Edwards et al. [4], Gore et al. [5], Harper et al. [6], Hills et al. [7], Hitchcock et al. [8], Hornery et al. [9], Ishiguro & Amano [10], Juliff et al. [11], Linseman et al. [12], Lynch et al. [13], Malan et al. [14], McCarthy et al. [15], Minett et al. [16], Naito et al. [17], Palmer et al. [18], Pan et al. [19], Price et al. [20], Yanci et al. [21] |
| **No valid core temperature measure (n = 4)** | Aragon-Vargas et al. [22], Herrera-Valenzuela et al. [23], Meir & Davie [24], Meir et al. [25] |
| **Conference abstract (n = 4)** | Bartolozzi & Godek [26], Sugiyama et al. [27], Taylor et al. [28], Taylor et al. [29] |
| **Youth athletes (n = 3)** | Christaras et al. [31] , Guerra et al. [32], Yeargin et al. [33] |
| **Core temperature not reported (n = 1)** | RicoSanz et al. [30] |
| **Athletes with injury (n = 1)** | Logan-Sprenger & Mc Naughton [34] |
| **Control only data not reported (n = 1)** | Bandelow et al. [35] |

**References**

1. Chalmers S, Siegler J, Lovell R, Lynch G, Gregson W, Marshall P, et al. Brief in-play cooling breaks reduce thermal strain during football in hot conditions. J Sci Med Sport. 2019;22(8):912-7.

2. Crowther RG, Leicht AS, Pohlmann JM, Shakespear-Druery J. Influence of rest on players' performance and physiological responses during basketball play. Sports (Basel). 2017;5(2).

3. Driscoll RL, McCarthy DG, Palmer MS, Spriet LL. Mild dehydration impaired intermittent sprint performance and thermoregulation in females. Appl Physiol Nutr Metab. 2020;45(9):1045-8.

4. Edwards AM, Mann ME, Marfell-Jones MJ, Rankin DM, Noakes TD, Shillington DP. Influence of moderate dehydration on soccer performance: physiological responses to 45 mm of outdoor match-play and the immediate subsequent performance of sport-specific and mental concentration tests. Br J Sports Med. 2007;41(6):385-91.

5. Gore CJ, Bourdon PC, Woolford SM, Pederson DG. Involuntary dehydration during cricket. Int J Sports Med. 1993;14(7):387-95.

6. Harper LD, Hunter R, Parker P, Goodall S, Thomas K, Howatson G, et al. Test-retest reliability of physiological and performance responses to 120 minutes of simulated soccer match play. J Strength Cond Res. 2016;30(11):3178-86.

7. Hills SP, Aben HGJ, Starr DP, Kilduff LP, Arent SM, Barwood MJ, et al. Body temperature and physical performance responses are not maintained at the time of pitch-entry when typical substitute-specific match-day practices are adopted before simulated soccer match-play. J Sci Med Sport. 2021;24(5):511-6.

8. Hitchcock KM, Millard-Stafford ML, Phillips JM, Snow TK. Metabolic and thermoregulatory responses to a simulated American football practice in the heat. J Strength Cond Res. 2007;21(3):710-7.

9. Hornery DJ, Farrow D, Mujika I, Young WB. Caffeine, carbohydrate, and cooling use during prolonged simulated tennis. Int J Sports Physiol Perform. 2007;2(4):423-38.

10. Ishiguro A, Amano T. Comparisons of cardiorespiratory and thermoregulatory responses to table tennis and cycling at similar perceived levels of effort. Sci Sports. 2022;37(2):143.e1-.e9.

11. Juliff LE, Peiffer JJ, Halson SL. Night games and sleep: physiological, neuroendocrine, and psychometric mechanisms. Int J Sports Physiol Perform. 2018;13(7):867-73.

12. Linseman ME, Palmer MS, Sprenger HM, Spriet LL. Maintaining hydration with a carbohydrate-electrolyte solution improves performance, thermoregulation, and fatigue during an ice hockey scrimmage. Appl Physiol Nutr Metab. 2014;39(11):1214-21.

13. Lynch GP, Periard JD, Pluim BM, Brotherhood JR, Jay O. Optimal cooling strategies for players in Australian Tennis Open conditions. J Sci Med Sport. 2018;21(3):232-7.

14. Malan M, Dawson B, Goodman C, Peeling P. Effect of heat exposure on thermoregulation and hockey-specific response time in field hockey goalkeepers. J Sci Med Sport. 2010;13(3):371-5.

15. McCarthy DG, Wickham KA, Vermeulen TF, Nyman DL, Ferth S, Pereira JM, et al. Impairment of Thermoregulation and Performance via Mild Dehydration in Ice Hockey Goaltenders. Int J Sports Physiol Perform. 2020;15(6):833-40.

16. Minett G, Duffield R, Kellett A, Portus M. Effects of mixed-method cooling on recovery of medium-fast bowling performance in hot conditions on consecutive days. J Sports Sci. 2012;30(13):1387-96.

17. Naito T, Sagayama H, Akazawa N, Haramura M, Tasaki M, Takahashi H. Ice slurry ingestion during break times attenuates the increase of core temperature in a simulation of physical demand of match-play tennis in the heat. Temperature (Austin). 2018;5(4):371-9.

18. Palmer MS, Heigenhauser G, Duong M, Spriet LL. Ingesting A Sports Drink Enhances Simulated Ice Hockey Performance While Reducing Perceived Effort. Int J Sports Med. 2017;38(14):1061-9.

19. Pan X, Song M, Pan X. Sports training to detect heart rate and body temperature in teenagers. Revista Brasileira de Medicina do Esporte. 2022;28(6):830-3.

20. Price MJ, Boyd C, Goosey-Tolfrey VL. The physiological effects of pre-event and midevent cooling during intermittent running in the heat in elite female soccer players. Appl Physiol Nutr Metab. 2009;34(5):942-9.

21. Yanci J, Iturricastillo A, Granados C. Heart rate and body temperature response of wheelchair basketball players in small-sided games. Int J Perform Anal Sport. 2014;14(2):535-44.

22. Aragón-Vargas L, Moncada-Jiménez J, Hernández-Elizondo J, Barrenechea A, Monge-Alvarado M. Evaluation of pre-game hydration status, heat stress, and fluid balance during professional soccer competition in the heat. Eur J Sport Sci. 2009;9(5):269-76.

23. Herrera-Valenzuela T, Ibieta C, Fuentes MS, Saez-Madain P, Lopez JC, Verdugo F, et al. Physiological responses of elite karate athletes during simulated competition. Ido Mov Culture J Martial Arts Anthrop. 2019;19(4):45-50.

24. Meir RA, Davie AJ, Ohmser P. Thermoregulatory responses of rugby league footballers playing in warm humid conditions. Sport Health. 1990;8(4):11-4.

25. Meir R, Brooks L, Shield T. Body weight and tympanic temperature change in professional rugby league players during night and day games: A study in the field. J Strength Cond Res. 2003;17(3):566-72.

26. Bartolozzi AR, Godek SF. Core Temperature in College Football Players during a Game Played in Hot Conditions. Med Sci Sports Exerc. 2006;38(5):S58-S.

27. Sugiyama K, Matsui T, Naito H. Effect of a water ingestion method on rectal temperature during a basketball game played in the heat. In Proceedings FISU/CESU Conference - The 18th Universiade 1995 Fukuoka, Japan - Sport and man: creating a new vision, 24, 25, 26 August 1995, Fukuoka, Organizing Committee for the Universiade 1995, c1995, p464-465.

28. Taylor L, Chrismas BCR, Stevens CJ, Coutts AJ, Henderson MJ. Elite female rugby sevens tournament match-play - core temperature changes. Med Sci Sports Exerc. 2020;52(17):968.

29. Taylor L, Thornton H, Lumley N, Stevens C. Game-to-game increases in core temperature during rugby 7's world series tournaments. Med Sci Sports Exerc. 2018;50(5):339-.

30. RicoSanz J, Frontera WR, Rivera MA, RiveraBrown A, Mole PA, Meredith CN. Effects of hyperhydration on total body water, temperature regulation and performance of elite young soccer players in a warm climate. Int J Sports Med. 1996;17(2):85-91.

31. Christaras M, Michailidis Y, Mandroukas A, Vardakis L, Christoulas K, Metaxas T. Effects of a short half-time re-warm-up program on matches running performance and fitness test performance of male elite youth soccer players. Appl Sci. 2023;13(4):2602.

32. Guerra I, Chaves R, Barros T, Tirapegui J. The influence of fluid ingestion on performance of soccer players during a match. J Sport Sci Med. 2004 Dec;3(4):198-202.

33. Yeargin SW, Dickinson JJ, Emerson DM, Koller J, Torres-McGehee TM, Kerr ZY. Exertional heat illness risk factors and physiological responses of youth football players. J Sport Health Sci. 2021;10(1):91-8.

34. Logan-Sprenger HM, Mc Naughton LR. Characterizing thermoregulatory demands of female wheelchair basketball players during competition. Res Sports Med. 2020;28(2):256-67.

35. Bandelow S, Maughan R, Shirreffs S, Ozgunen K, Kurdak S, Ersoz G, et al. The effects of exercise, heat, cooling and rehydration strategies on cognitive function in football players. Scand J Med Sci Sports. 2010;20:148-60.
