# Supplementary material for: Core Body Temperatures in Intermittent Sports: A Systematic Review
Source: Sports Med. 2023 Aug 1;53(11):2147–70. doi: 10.1007/s40279-023-01892-3 (PMC10587327; doi:10.1007/s40279-023-01892-3)
Supplement: Supplementary file 6 — Supplementary file6 (DOCX 48 KB) [file 40279_2023_1892_MOESM6_ESM.docx]

#
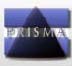
PRISMA 2020 Checklist

*From:* Page MJ, McKenzie JE, Bossuyt PM, Boutron I, Hoffmann TC, Mulrow CD, et al. The PRISMA 2020 statement: an updated guideline for reporting systematic reviews. BMJ 2021;372:n71. doi: 10.1136/bmj.n71

For more information, visit: <http://www.prisma-statement.org/>

**Table 1.** Explanation of how each item was addressed including, where possible, the text used in the manuscript.

| **Section and topic** | **Item**  **#** | **How each item was addressed** |
| --- | --- | --- |
| **TITLE** | | |
| Title | 1 | Core body temperature in intermittent sports: A systematic review |
| **ABSTRACT** | | |
| Abstract | 2 | Each element of the PRISMA for Abstracts is specifically addressed within the abstract of the manuscript in the relevant sections (although where the PRISMA titled a section “Discussion”, I’ve put these elements in my “Conclusion” section). |
| **INTRODUCTION** | | |
| Rationale | 3 | Provided on lines 105 – 116.  “Quantifying the highest thermal strain (i.e., peak Tc) from a range of intermittent sports would enhance our understanding of the thermal requirements of sport and assist in making informed decisions about training or match-day interventions to reduce thermally-induced harm and/or performance decline. The efficacy of applied heat acclimation/acclimatisation training interventions [27] and acute mixed-method cooling protocols [21] is supported by a considerable body of evidence [74]. Therefore, with increasing globalisation in sport enabling year-round competition in warmer climates and the ongoing effects of climate change [75], best practice management of exercise-induced hyperthermia (through targeted application of these interventions) will be of increasing importance. Identifying appropriate action for athletes and support staff should be informed by available peer-reviewed literature, and currently, no reviews of the literature provide a synthesis of the thermal strain data collected in-competition during intermittent sports. Further, increased understanding of the magnitude of thermal strain in competing athletes could be used to guide policy surrounding thermoregulatory health and safety at sporting events.” |
| Objectives | 4 | Provided in the last sentence of the fourth paragraph of the introduction (lines 117 – 119).  “The purpose of this review is to provide athletes, practitioners, and policy makers a synthesis of the thermal strain literature to determine the need for interventions aimed at mitigating exercise-induced hyperthermia in intermittent sport athletes.” |
| **METHODS** | | |
| Eligibility criteria | 5 | Provided in the Eligibility Criteria section (lines 134 – 145).  “Studies were considered eligible if they included healthy athletes competing in intermittent sports competition at any level. Non-human subjects, youth athletes (study participants’ mean age minus 2 standard deviations is less than 16 years), or participants with chronic disease, disability, metabolic disorders, or injury were excluded due to differences in the physiological responses to sports. Interventions aimed at both adult and youth athletes were included only if the data provided for adults was reported separately. All exposures including athletes involved in intermittent sport competition (competitive, friendly, or experimental) played within normal parameters (e.g., field size, playing numbers) were included in this review. Outcomes of interest included only internally measured Tc (i.e., gastrointestinal or rectal; shown to display acceptable agreement [78]). No limitations were placed on the study design if the intervention met the eligibility criteria. Studies were included only if Tc was measured during competition or breaks in play and without experimental intervention that may influence thermal strain (e.g., cooling).” |
| Information sources | 6 | Provided in the Search section (lines 149 – 151).  “A literature search was conducted by one author (MH) in the electronic bibliographic databases of Web of Science Core Collection, Ovid MEDLINE, and EBSCOhost SPORTDiscus. Databases were searched from inception up until April 2023.” |
| Search strategy | 7 | Presented in supplementary material 2. |
| Selection process | 8 | Provided in Study Selection section (lines 167 – 174).  “Articles retrieved through the systematic search were exported into a reference management software (EndNote version X8) and all duplicate articles were removed. All references were then imported into Covidence (Covidence Systematic Review Software, Veritas Health Innovation, 2013) for assessment of eligibility. Two authors (MH, CG) independently screened the records by title and abstract, with all potentially eligible references proceeding to full-text screening with conflicts resolved by a third author (FMI). Authors (MH, CG) then independently screened the full text of all included articles against the eligibility criteria. Interrater reliability, as measured by Cohen’s Kappa (κ), was 0.75 during the title and abstract screening and 0.85 during full-text screening.” |
| Data collection process | 9 | Provided in Data Extraction section (lines 178 – 179).  “Data was extracted by two authors (MH, CG) and imported into an Excel spreadsheet created for this review (supplementary material 3). Extracted data were compared with any discrepancies resolved through discussion.” |
| Data items | 10a | Provided in Data Extraction section (lines 180 – 183).  “Information extracted from each eligible study included publication details (author, year), participant characteristics (sex, level of competition, sample size), study methods (design, types of measurement, recording frequency), exposure (sport, competition type, duration, environmental conditions, location of data collection, home location of participants), and outcome (core body temperature).” |
|  | 10b | Provided in Data Extraction section (lines 180 – 183).  “Information extracted from each eligible study included publication details (author, year), participant characteristics (sex, level of competition, sample size), study methods (design, types of measurement, recording frequency), exposure (sport, competition type, duration, environmental conditions, location of data collection, home location of participants), and outcome (core body temperature).” |
| Study risk of bias assessment | 11 | Provided in Risk of Bias section (lines 224 - 228).  “The current review focused on a specific measure (Tc) as measured in a control (non-experimentally manipulated) condition. Accordingly, no available tool, to our knowledge, specifically includes potential sources of bias for physiological responses in such a context. We, therefore, did not examine the risk of bias but instead developed and used a methodological evaluation checklist to document what we deemed to be important methodological considerations for researchers conducting future investigations.” |


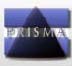
**PRISMA 2020 Checklist**

| Effect measures | 12 | The effect measure in the present review is peak core body temperature. This is explained in the final sentence of section 2.4 (Data Extraction): “Information extracted from each eligible study included publication details (author, year), participant characteristics (sex, level of competition, sample size), study methods (design, types of measurement, recording frequency), exposure (sport, competition type, duration, environmental conditions, location of data collection, home location of participants), and outcome (T_c_).” |
| --- | --- | --- |
| Synthesis methods | 13a | The process for determining eligible studies for synthesis is explained in the Data Synthesis section:  “All studies and group conditions that reported parametric measures of centrality and variability (mean and standard deviation) were included in this synthesis.” on lines 201-203.  Also, on lines 209-211: “All studies that provided (1) competition duration and (2) WBGT or ambient temperature and relative humidity were considered eligible for this synthesis”. |
|  | 13b | Methods used to prepare the data for synthesis are available on lines 197 – 201:  “Standard deviations for Tc (reported in text or extracted from figures) were converted to standard errors by dividing by the square root of the sample size. Standard errors were subsequently converted to confidence intervals by multiplying by the Z-value associated with the desired level of confidence (Z = 0.674, 1.282, 1.960, and 2.576 for 50, 80, 95, and 99% confidence intervals, respectively). Finally, adding or subtracting the resulting values from the mean provided upper or lower confidence limits.”  Also, on line 203-204: “Medians and interquartile ranges reported in Stay et al. [82] were transformed to estimated means and standard deviations using the method outlined in Luo et al. [83]”.  Also, on lines 211-212: “Nine studies did not report WBGT, so estimates were calculated using the validated Liljegren method [84]”. |
|  | 13c | Methods of displaying results of individual studies are explained on lines 190-191:  “Data were therefore presented descriptively in tabular format and graphically.”  Figure captions provide additional information regarding the visual presentation of the results. |
|  | 13d | Data were not meta-analysed given the methodological heterogeneity between studies (including differing core temperature measurements, environmental conditions, and exposure durations to a variety of sports). We anticipated based on a scoping search that the heterogeneity could not be explored given that sub-group analyses would leave too few studies in each group for investigating the different moderators. Data were therefore presented descriptively in tabular and graphical format. Although a meta-analysis was not planned (or pre-registered) for the aforementioned reasons, summary estimates and forest plots of overall and subgroup meta-analyses have been provided at the request of a reviewer (supplementary material 4). As expected, high heterogeneity was found in both the overall and subgroup analyses. |
|  | 13e | Same as item #13d. |
|  | 13f | Same as item #13d. |
| Reporting bias assessment | 14 | Not applicable. Risk of bias was not performed as explained in item #11. |
| Certainty assessment | 15 | No GRADE was applied given the main outcome was not an effect of treatment/intervention. |
| **RESULTS** | | |
| Study selection | 16a | PRISMA flow chart in Figure 1. Also discussed in text in Study Selection section (lines 251 – 256). |
|  | 16b | Provided in supplementary material 4 and written in text on lines 251 – 256.  “A total of 68 studies were retrieved as full text and assessed for eligibility (one report not retrieved [89]), and of those, 35 were excluded (reasons for the exclusion provided in Figure 1 and supplementary material 5).” |
| Study characteristics | 17 | Table 1. |
| Risk of bias in studies | 18 | As discussed above, a methodological evaluation checklist was used rather than assessing risk of bias due to the nature of the studies included in this review. The results of the methodological evaluation for each study are available in Table 2. |
| Results of individual studies | 19 | Results of individual studies are presented in Table 1 (all studies), Figure 4, and Figure 5 (all studies that reported (1) competition duration, and (2) either WBGT or ambient temperature and relative humidity). |
| Results of syntheses | 20a | Summaries of the relevant study characteristics is provided in section 3.4 and methodological evaluation in section 3.6. |
|  | 20b | Same as item #13d. |
|  | 20c | Same as item #13d. |
|  | 20d | Same as item #13d. |


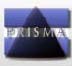
**PRISMA 2020 Checklist**

| Reporting biases | 21 | Not applicable. Risk of bias was not performed as explained in item #11. |
| --- | --- | --- |
| Certainty of evidence | 22 | No GRADE was applied given the main outcome was not an effect of treatment/intervention. |
| **DISCUSSION** | | |
| Discussion | 23a | Provided in section 4.1 Summary of main results |
|  | 23b | Provided in section 4.2 Quality of the evidence |
|  | 23c | Provided in section 4.3 Limitations and potential biases in the review process |
|  | 23d | Provided in section 4.4 Implications for practice and future research |
| **OTHER INFORMATION** | | |
| Registration and protocol | 24a | A systematic review protocol that included the review question, search strategy, and exclusion criteria was registered with the Open Science Framework (<https://osf.io/vfb4s>; DOI: 10.17605/OSF.IO/EZYFA, 4th January 2021). |
|  | 24b | The protocol can be accessed at <https://osf.io/vfb4s> or through DOI: 10.17605/OSF.IO/EZYFA. |
|  | 24c | In section 9e of the registration, the outcome of interest was stated to be “any reported measure of player peak Tc”. This was later refined to internally measured core temperature measurements only (gastrointestinal or rectal), as these measures have displayed acceptable validity, reliability, and agreement.  In section 13 of the registration, the modified Appraisal tool for Cross‐Sectional Studies (AXIS) was stated as the instrument that would be used to critically appraise bias in the study design. As a result of the characteristics of the studies returned from our systematic search strategy (descriptive), this was no longer appropriate to use. No available tool, to our knowledge, specifically includes potential sources of bias for physiological responses in such a context. We, therefore, developed and used a methodological evaluation checklist to document what we deemed to be important methodological considerations for researchers conducting future investigations. This can be found in section 2.6.1 of the manuscript. |
| Support | 25 | Reported according to the journal requirement. MH is supported by the Australian Government’s Research Training Program scholarship. There were no other funders or sponsors for this review. |
| Competing interests | 26 | Any competing interests of review authors declared (none). |
| Availability of data, code and other materials | 27 | Data extracted from included studies is available in supplementary material 3. The R code used to develop the search string is available in PDF format (with code explanation) in supplementary material 1 or in an R script available at <https://osf.io/xam5v>. The bibliographic data (returned from the systematic search) required to reproduce the analysis in the R code is available in multiple files within the Open Science Framework repository associated with this review (<https://osf.io/ezyfa/>). |

**Table 2.** Details of the requirements for each item.

| **Section and Topic** | **Item #** | **Checklist item** |
| --- | --- | --- |
| **TITLE** | | |
| Title | 1 | Identify the report as a systematic review. |
| **ABSTRACT** | | |
| Abstract | 2 | See the PRISMA 2020 for Abstracts checklist. |
| **INTRODUCTION** | | |
| Rationale | 3 | Describe the rationale for the review in the context of existing knowledge. |
| Objectives | 4 | Provide an explicit statement of the objective(s) or question(s) the review addresses. |
| **METHODS** | | |
| Eligibility criteria | 5 | Specify the inclusion and exclusion criteria for the review and how studies were grouped for the syntheses. |
| Information sources | 6 | Specify all databases, registers, websites, organisations, reference lists and other sources searched or consulted to identify studies. Specify the date when each source was last searched or consulted. |
| Search strategy | 7 | Present the full search strategies for all databases, registers and websites, including any filters and limits used. |


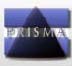
**PRISMA 2020 Checklist**

| **Section and Topic** | **Item #** | **Checklist item** |
| --- | --- | --- |
| Selection process | 8 | Specify the methods used to decide whether a study met the inclusion criteria of the review, including how many reviewers screened each record and each report retrieved, whether they worked independently, and if applicable, details of automation tools used in the process. |
| Data collection process | 9 | Specify the methods used to collect data from reports, including how many reviewers collected data from each report, whether they worked independently, any processes for obtaining or confirming data from study investigators, and if applicable, details of automation tools used in the process. |
| Data items | 10a | List and define all outcomes for which data were sought. Specify whether all results that were compatible with each outcome domain in each study were sought (e.g. for all measures, time points, analyses), and if not, the methods used to decide which results to collect. |
|  | 10b | List and define all other variables for which data were sought (e.g. participant and intervention characteristics, funding sources). Describe any assumptions made about any missing or unclear information. |
| Study risk of bias assessment | 11 | Specify the methods used to assess risk of bias in the included studies, including details of the tool(s) used, how many reviewers assessed each study and whether they worked independently, and if applicable, details of automation tools used in the process. |
| Effect measures | 12 | Specify for each outcome the effect measure(s) (e.g. risk ratio, mean difference) used in the synthesis or presentation of results. |
| Synthesis methods | 13a | Describe the processes used to decide which studies were eligible for each synthesis (e.g. tabulating the study intervention characteristics and comparing against the planned groups for each synthesis (item #5)). |
|  | 13b | Describe any methods required to prepare the data for presentation or synthesis, such as handling of missing summary statistics, or data conversions. |
|  | 13c | Describe any methods used to tabulate or visually display results of individual studies and syntheses. |
|  | 13d | Describe any methods used to synthesize results and provide a rationale for the choice(s). If meta-analysis was performed, describe the model(s), method(s) to identify the presence and extent of statistical heterogeneity, and software package(s) used. |
|  | 13e | Describe any methods used to explore possible causes of heterogeneity among study results (e.g. subgroup analysis, meta-regression). |
|  | 13f | Describe any sensitivity analyses conducted to assess robustness of the synthesized results. |
| Reporting bias assessment | 14 | Describe any methods used to assess risk of bias due to missing results in a synthesis (arising from reporting biases). |
| Certainty assessment | 15 | Describe any methods used to assess certainty (or confidence) in the body of evidence for an outcome. |
| **RESULTS** | | |
| Study selection | 16a | Describe the results of the search and selection process, from the number of records identified in the search to the number of studies included in the review, ideally using a flow diagram. |
|  | 16b | Cite studies that might appear to meet the inclusion criteria, but which were excluded, and explain why they were excluded. |
| Study characteristics | 17 | Cite each included study and present its characteristics. |
| Risk of bias in studies | 18 | Present assessments of risk of bias for each included study. |
| Results of individual studies | 19 | For all outcomes, present, for each study: (a) summary statistics for each group (where appropriate) and (b) an effect estimate and its precision (e.g. confidence/credible interval), ideally using structured tables or plots. |
| Results of syntheses | 20a | For each synthesis, briefly summarise the characteristics and risk of bias among contributing studies. |
|  | 20b | Present results of all statistical syntheses conducted. If meta-analysis was done, present for each the summary estimate and its precision (e.g. |


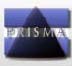
**PRISMA 2020 Checklist**

| **Section and Topic** | **Item #** | **Checklist item** |
| --- | --- | --- |
|  |  | confidence/credible interval) and measures of statistical heterogeneity. If comparing groups, describe the direction of the effect. |
|  | 20c | Present results of all investigations of possible causes of heterogeneity among study results. |
|  | 20d | Present results of all sensitivity analyses conducted to assess the robustness of the synthesized results. |
| Reporting biases | 21 | Present assessments of risk of bias due to missing results (arising from reporting biases) for each synthesis assessed. |
| Certainty of evidence | 22 | Present assessments of certainty (or confidence) in the body of evidence for each outcome assessed. |
| **DISCUSSION** | | |
| Discussion | 23a | Provide a general interpretation of the results in the context of other evidence. |
|  | 23b | Discuss any limitations of the evidence included in the review. |
|  | 23c | Discuss any limitations of the review processes used. |
|  | 23d | Discuss implications of the results for practice, policy, and future research. |
| **OTHER INFORMATION** | | |
| Registration and protocol | 24a | Provide registration information for the review, including register name and registration number, or state that the review was not registered. |
|  | 24b | Indicate where the review protocol can be accessed, or state that a protocol was not prepared. |
|  | 24c | Describe and explain any amendments to information provided at registration or in the protocol. |
| Support | 25 | Describe sources of financial or non-financial support for the review, and the role of the funders or sponsors in the review. |
| Competing interests | 26 | Declare any competing interests of review authors. |
| Availability of data, code and other materials | 27 | Report which of the following are publicly available and where they can be found: template data collection forms; data extracted from included studies; data used for all analyses; analytic code; any other materials used in the review. |
